# Changes in Fertility Trends and Women’s Fertility Desires in the Wake of the Homicide Surge in Mexico

**DOI:** 10.1007/s11113-026-09999-x

**Published:** 2026-03-16

**Authors:** Ginevra Floridi, Maria Gargiulo, José Manuel Aburto

**Affiliations:** 1 School of Social and Political Science, University of Edinburgh, Chrystal Macmillan Building, 15a George Square, Edinburgh, EH8 9LD UK; 2 Department of Population Health, London School of Hygiene and Tropical Medicine, Keppel Street, WC1E 7HT London, UK

**Keywords:** Violence, Total fertility rate, Fertility desires, Birth rates

## Abstract

Since 2006, Mexico has experienced a surge in homicides due to national policies and international influences on drug trafficking activities. Although the effects of the so-called “Drug War” have been extensively studied in demography and social science research, whether and how the increase in homicides has affected fertility is poorly understood. This study provides a comprehensive account of the association between homicides and changes in fertility rates and desires in Mexico. Using population-level administrative data on births, deaths, and homicides for 2443 municipalities, we apply fixed-effects models and a staggered difference-in-differences estimator to study the effect of homicidal violence on total fertility rate (TFR), crude birth rate (CBR), and birth counts across Mexican municipalities between 2000 and 2020. Then, using random-intercept and fixed-effects models, we analyze the association between changes in homicide rates and fertility desires for 6341 women from the Mexican Family Life Survey (2002–2012). Our findings show very small negative associations, and no overall effect of homicides on fertility for the period considered. Similarly, we find no association between municipality-level homicide rates and fertility desires, consistently by education, age, and parity. Our results show remarkable continuity in the Mexican fertility decline despite the rapid escalation of violence.

**Supplementary Information:**

The online version contains supplementary material available at 10.1007/s11113-026-09999-x.

## Introduction

Exposure to violence can have significant implications for individual livelihoods and populations. Violence affects the determinants of demographic change, namely mortality (Aburto et al., 2016), fertility (Cetorelli, 2014), and migration (Abel et al., 2019). This paper examines the effect of increases in the homicide rate on fertility rates and women’s fertility desires. We focus on Mexico, where the homicide rate rapidly increased to very high levels in the past two decades. Studying changes in individual fertility desires alongside area-level summary indicators of fertility allows us to delve deeper into the mechanisms by which homicidal violence may affect demographic trends. From a policy perspective, it can reveal potential mismatches between desired family size and actualized behavior that call for interventions to expand the coverage of family planning services in the areas most affected by violence (Svallfors, 2022).

Since 2006, Mexico has undergone an unprecedented wave of homicidal violence due to national policies and international influences on drug trafficking activities, the so-called “Drug War” (Dell, 2015). This has affected life expectancy (Aburto et al., 2016), union formation and dissolution (Caudillo and Lee 2023a), infant health (Brown, 2018; Chang et al., 2024), mental health (Balmori de la Miyar, 2020; Romero-Gómez et al., 2024; Villarreal & Yu, 2017), and socio-economic outcomes (Balmori de la Miyar, 2016; Brown & Velásquez, 2017; Carrasco & Durán-Bustamante, 2018; Velásquez, 2019). The effects of violence exposure tend to differ by gender (Cockburn, 2004). In the case of the “Drug War,” the impact on mortality and educational attainment has been particularly pronounced among young males (Aburto et al., 2016; Brown & Velásquez, 2017), but the psychological toll of rising homicides may fall disproportionately on women through the disruption of everyday life (Cockburn, 2004). The psychological and structural changes brought about by homicides may have influenced fertility behaviors and preferences. However, we do not know how the increase in homicides affected fertility rates nor desires among women of reproductive age.

We investigate the effect of rising homicide rates on total fertility rate (TFR) across Mexican municipalities, and repeat the analysis for the crude birth rate (CBR) as well as birth counts. We also study changes in fertility desires among women of reproductive age. In the first part of the analysis, we document changes in fertility since 2000, and study the association between homicide rates and TFR across 2,443 municipalities. Using fixed-effects models and a staggered difference-in-differences (DID) estimator, we find very small to no reductions in TFR subsequent to rising homicides. We find similar results when using the CBR and birth counts as indicators of fertility. In the second part of the analysis, we study the association between municipality-level homicide rates and changes in fertility desires between 2002 and 2012 using survey data. Models with woman-specific random intercepts and fixed effects show no association between homicides and fertility desires. Our study consistently points to unchanged fertility in the wake of the homicide surge, contributing to a comprehensive understanding of the impact of homicides in Mexico.

## Background

### Fertility Trajectories in Mexico, 1960s–2020s

The fertility transition in Mexico since the 1960s mirrored the pattern of rapid fertility decline observed in other Latin American countries including Colombia, Paraguay, and Brazil (Castro Torres, 2021). According to United Nations estimates, Mexico experienced its highest recorded TFR in the early 1960s, at a level of 6.8 children per woman (UN, 2022). Fertility decline in Mexico unfolded over three phases (Tuiran et al., 2002). During an “initial descent” (1964–1973), the TFR decreased from 6.8 to 6.3 children per woman. The years between 1974 and 1984 were characterized by an “accelerated descent” after the creation of the *Consejo Nacional de Población* (National Population Council, CONAPO), aimed at expanding access to family planning services (Rodriguez-Barocio et al., 1980). Over this phase, the TFR declined to around 4.2 children per woman. This was followed by a prolonged “moderate decline,” with the TFR stabilizing around 2.5 children per woman in the early 2000’s and reaching replacement levels (2.1) in 2015 (UN, 2025). Throughout this period (2003–2020), the implementation of public healthcare coverage through the *Seguro Popular* program contributed to improved infant health (Kumar & Gonzalez, 2018), reduced marriage rates, and higher cohabitation among poorer households (Azuara, 2011), although it is unclear whether and how it affected fertility. Since 2016, the TFR has been consistently below replacement level, reaching 1.99 in 2020 and expected to reach 1.87 by 2025 (UN, 2025). During the COVID-19 pandemic, fertility declined temporarily, with a fall in the general fertility rate by 12%, followed by a rebound to pre-pandemic levels by the end of 2021 (Silverio-Murillo et al., 2024). Relatively less is known about sub-national trends in fertility. Over the last half century, Mexico has undergone important economic, social, cultural and environmental changes—including the increase in homicides—that may affect fertility, but these have occurred at different pace across areas (Páez & Zavala de Cosío, 2017).

Fertility decline in Mexico has been socio-economically stratified across individuals (Castro Torres, 2021; Páez & Zavala de Cosío, 2017) and states (Tuiran et al., 2002). Women of higher socio-economic status delayed the timing of their first birth and mainly achieved smaller family sizes through non-permanent contraceptive methods, while women of lower socio-economic status anticipated the timing of first birth and stopped having children early, often via sterilization (Castro Torres, 2021). At the state level, fertility decline began in the most economically advantaged states, but quickly extended to the rest (Tuiran et al., 2002). The fastest states to undergo the transition to low birth rates include Mexico City and the northern states of Baja California, Baja California Sur, Coahuila, Nuevo León, Sonora, and Tamaulipas. The last states to attain a low fertility regime are mostly in the South: Chiapas, Guerrero, Michoacán, San Luis Potosí, and Oaxaca, as well as Zacatecas in the North (Tuiran et al., 2002). At the municipality level, around 47% of municipalities had below-replacement fertility in 2020 (Núñez Medina, 2022). However, municipalities in rural areas without access to health services have experienced slower declines in TFR (Núñez Medina, 2022). These trends have not been previously linked to the increase in homicides. In this paper, we document state- and municipality-level changes in fertility rates between 2000 and 2020 in Mexico, and link them to homicide rates as well as indicators of socio-economic deprivation.

### The Surge in Homicides in Mexico

**Fig. 1:**
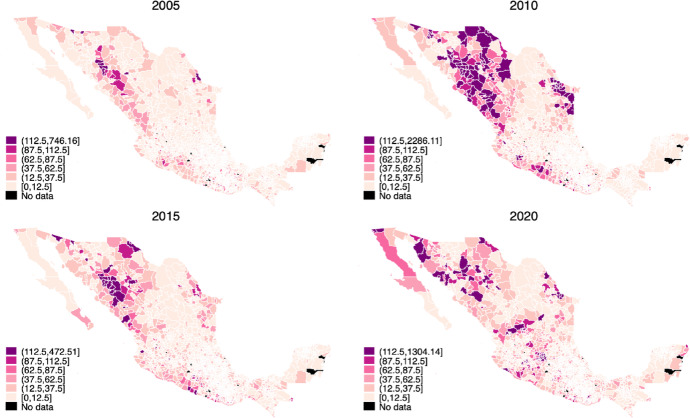
Distribution of homicides per 100,000 inhabitants across Mexican municipalities, 2005–2020

Homicide rates have dramatically increased in Mexico since December 2006. To put this into perspective, the homicide rate more than doubled between 2007 and 2011, from 8.4 to 23.7 homicides per 100,000 population in 2011 (Canudas-Romo et al., 2017). The stark rise in homicides and other violent crimes since 2006 has been widely documented and analyzed (Brown, 2018; Dell, 2015; Guerrero-Gutierrez, 2011; Rios, 2013). The general consensus about its causes is centered around the so-called “Drug War” first implemented by former president Felipe Calderón, which involves the direct intervention of the military to crack down on drug trafficking organizations. Military capture or killing of drug trafficking organization leaders has resulted in the fracturing of the original organizations and intense within-group fighting over power vacuums (Guerrero-Gutierrez, 2011). Estimates suggest that over 85% of homicides have involved drug traffickers killing each other (Dell, 2015). Violent crimes toward civilians also increased as traffickers turned to extortion, kidnapping, human smuggling, arson, and car theft due to lower revenues from trafficking activities (Dell, 2015; Guerrero-Gutierrez, 2011). In the early stages of the “Drug War,” homicides remained concentrated in the most valuable regions for drug trafficking organizations to control, with the states of Michoacán, Guerrero, Sinaloa, and Chihuahua accounting for the majority of killings in 2007 (Espinal-Enríquez & Larralde, 2015; Rios, 2013). Since then, homicides spread first along the Pacific coast, and then to the northwestern part of the country, to the states of Nuevo León and Tamaulipas (Espinal-Enríquez & Larralde, 2015). Figure 1 compares the homicide rate per 100,000 people across Mexican municipalities in 2005, 2010, 2015, and 2020, showing wide spatial and time variation in homicides since the beginning of the “Drug War.”

It is unclear whether and how the “Drug War” has affected fertility. So far, two studies on infant health outcomes have yielded mixed evidence. Brown (2018) found that birth rates responded negatively to violence exposure during the middle of gestation for higher-educated women, and significantly declined for women in poorer health who were exposed to violence in early gestation. In contrast, Torche and Villarreal (2014) found no overall fertility response and minimal selectivity of women giving birth in the wake of rising homicides. A study of teenage pregnancies found that the average increase in municipal-level homicides corresponded to a decline in teenage pregnancies by 1.5%, mostly attributable to changes in the sexual behavior of young women—with exposure to violent crime at municipality level reducing the probability of ever having had sex (Tsaneva & Gunes, 2020). None of these studies address the question of changes in the TFR or fertility desires in the wake of the rapid increase in homicides.

## The Association Between Violence and Fertility

At the population level, exposure to violence through war or other forms of armed conflict can affect fertility rates by influencing the proximate and distal determinants of fertility. Among the proximate determinants, generalized violence can reduce opportunities for marriage and cohabitation as well as the stability of unions (Caudillo and Lee 2023a, b), and impair women’s fecundity through stress and health deterioration (Agadjanian & Prata, 2002; Thiede et al., 2020). The 1998–2000 conflict between Eritrea and Ethiopia led to a steep decline in fertility, largely by reducing the proportion of married women living with their husbands (Blanc, 2004; Woldemicael, 2008). Thiede et al. (2020) examined the association between exposure to armed conflict and fertility in 25 sub-Saharan African countries and found modest reductions in the probability of childbearing, suggesting this was mainly driven by reductions and delays in marriage and cohabitation. With regard to women’s health, O’Brien (2020) found that exposure to conflict during the Tajik civil war increased the likelihood of experiencing a miscarriage. By contrast, in the context of the Iraq War of 2003–2011, Cetorelli (2014) found an increase in fertility was due to anticipation of marriage and first birth during conflict years.

Exposure to violence can alter the structural determinants of fertility, by damaging infrastructure and affecting business cycles. Conflict and unrest may affect access to contraception and family planning services, resulting in increases in unplanned fertility. Svallfors and Billingsley (2019) studied the Colombian armed conflict and found lower contraceptive use in areas more affected by violence, which was not fully explained by higher fertility desires. Within the same context, Svallfors (2021) found increased uptake in sterilization in areas most affected by violence, suggesting that women may opt for sterilization due to the decreased accessibility of reversible methods of contraception. Conflict is also related to negative economic conditions (Velásquez, 2019). To the extent that economic crises reduce fertility (Goldstein et al., 2013), exposure to violence at area level may lower fertility rates. In the Latin American context, Adserà and Menendez (2011) documented decreased childbearing during economic downturns between the 1980s and the 2010s.

Exposure to violence has psychological consequences that may affect fertility rates at the population level (Caldwell, 2006; Villarreal & Yu, 2017). Generalized violence may lead families to have more children in the face of heightened mortality conditions (Rahman, 1998). Research on the Rwandan genocide found strong evidence for a “child replacement” effect, as having experienced the death of a child during conflict was related to higher hazards of having a child and more births in the 15 years after the conflict (Kraehnert et al., 2019; Schindler & Brück, 2011). Torrisi (2020) studying fertility changes in Azerbaijan found substantially higher probabilities of transitioning to second birth for women exposed to conflict relative to non-exposed women. However, fertility responses to violence through psychological pathways can also be negative. Caldwell (2006) argued that major social upheavals lead to increased uncertainty about the future and a desire to postpone childbirth until the situation is clearer. Recent studies of economic insecurity (Vignoli et al., 2020) and the COVID-19 pandemic (Guetto et al., 2022) found these to be related to fertility decline in the face of increased collective uncertainty. Finally, conflict can induce changes in fertility rates through forced or voluntary migration (Fargues, 2000). In Burundi, Verwimp et al. (2020) found the risk of a first pregnancy to be higher in the year in which a woman is forcibly displaced due to civil war, and lower in the year when a woman migrates voluntarily.

The general message from the empirical literature on the association between violence and fertility is one of heterogeneity. First, fertility responses to violence exposure are heterogeneous by the nature of the conflict being studied. Genocides (e.g., in Rwanda) and prolonged political conflicts (e.g., in Palestine) are generally associated with higher fertility (Fargues, 2000; Kraehnert et al., 2019). Studies of other types of social upheaval as well as outright war show evidence of fertility decline (Blanc, 2004; Caldwell, 2006), although the results are mixed (Cetorelli, 2014; Torrisi, 2020). A second source of heterogeneity is the time frame considered. In a review of evidence on crises, conflict, and displacement across different historical and geographical contexts, Hill (2004) found that violence tends to lower fertility in the short-run, but the effects vanish in the long-run. In a study of conflict in Angola, Agadjanian and Prata (2002) also found support the idea of a short-term fertility decline followed by a rebound. Heterogeneity in effects can also derive from the stage of the fertility transition of the affected population (Caldwell, 2006). For example, at the time when war with Ethiopia broke out, Eritrea was already undergoing a fertility decline, which may have been accelerated by conflict (Woldemicael, 2008). Finally, there is likely to be variation by the extent of violence exposure. Castro Torres and Urdinola (2019) examined the effect of the Colombian internal conflict on fertility and found a positive effect for rural areas only—which were the most affected by violence.

Given the lack of research on the association between violence exposure and fertility in the context of the Mexican “Drug War,” we deem it important to document fertility responses to this protracted and ongoing internal conflict that continues to affect demographic, psychological and socio-economic outcomes of the population (Aburto et al., 2016; Velásquez, 2019; Villarreal & Yu, 2017). We focus on the homicide rate, which is widely used to measure and compare levels of violence in Latin America (Rivera, 2016). Although other types of violence have also increased in Mexico since 2006 (Dell, 2015), homicides are the most commonly used measure in the literature on the effects of the so-called “Drug War” because they are less prone to measurement error and underreporting than other statistics about crime and violence, and are available from population death registers at highly granular (municipality) level for a long time series (Brown 2018; Caudillo and Lee 2023a). The first objective of our study is to assess how rising homicide rates affected fertility rates across Mexican municipalities between 2000 and 2020.

Based on the above discussion of mechanisms, we expect to observe a negative association between homicides and fertility in Mexico. Among the proximate determinants, the “Drug War” reduced the availability of male partners (Aburto et al., 2016) and increased the risk of union dissolution (Caudillo and Lee 2023a). Among the distal determinants, rising homicides have been associated with deteriorating socio-economic conditions (Velásquez, 2019). Moreover, given that fertility decline was well under way in Mexico in the mid-2000s (Núñez Medina, 2022), the increase in violence may have accelerated the decline (Woldemicael, 2008). Thus, we hypothesize that:

### H1

There is a negative association between homicides and fertility rates across Mexican municipalities between 2000 and 2020.

### Violence Exposure and Fertility Desires

Fertility desires are the first conscious expression of fertility preferences, and lead to intentions on whether or not to have children, parity, and the timing of childbearing. Within fertility preferences, desires are more distal determinants of fertility as they are based on someone’s feelings, whereas intentions are more proximate as they imply a commitment to act and are often based on a person’s current situation (Miller, 2011). Although the literature commonly refers to fertility intentions, most surveys measure desires by asking individuals how many (more) children they would like to have (Kost & Zolna, 2019). In this study we focus on fertility desires in line with the available survey questions.

Even though levels of over- and under-achieved fertility are high in low- and middle-income countries, fertility desires are predictive of realized fertility (Yeatman et al., 2020; Kodzi et al., 2010). At the same time, fertility desires are flexible (Yeatman et al., 2013). They respond to contingencies, inputs, and shifts at the micro and macro levels, rather than emerging from clarity about a predictable future (Johnson-Hanks et al., 2011; Ní Bhrolcháin & Beaujouan, 2019; Vignoli et al., 2020). Flexibility in childbearing is a strategic response to life’s uncertainties and it is especially important in contexts of political and social unrest (Johnson-Hanks, 2005). Fertility desires tend to vary according to individual characteristics. Previous literature identifies age, partnership status, employment status, and household socio-economic status as determinants of fertility preferences. Moreover, women’s fertility desires may depend on their own health as well as the total number of people living in the household (Buber-Ennser et al., 2024; Nobles et al., 2015; Svallfors, 2021; Zimmerman et al., 2022). Trinitapoli and Yeatman (2018) found that women’s fertility preferences in Malawi were related to existential uncertainty, which was proxied by having ever experienced a miscarriage or the death of a child, a death in the close network, and a heightened sense of one’s own mortality. The unexpected onset of violence may shape women’s fertility preferences by increasing the perceived (as well as actual) unpredictability of lives (Caldwell, 2006).

In line with insurance and replacement mechanisms (Rahman, 1998), women may desire more children subsequent to increased homicides exposure in the hope that at least some of those children will survive. Rutayisire et al. (2013) found increased fertility preferences in the wake of the Rwandan genocide. In a study of women aged 18–22 in the U.S. state of Michigan, Weitzman et al. (2021) found that nearby homicides have a positive, long-term effect on young women’s desire for pregnancy, which emerged three-to-five months post exposure to violence. On the other hand, witnessing violence may discourage women from having children by lowering their expectation that any unborn child will have a good life (Agadjanian, 2005). Studies in high-income countries suggest that uncertainty related to economic downturn, environmental issues, and the COVID-19 pandemic have resulted in lower desired family size (Lazzari et al., 2024; Rackin et al., 2022; Vignoli et al., 2020). Some research instead indicates stability in fertility preferences in the wake of uncertainty. Buber-Ennser et al. (2024) found no changes in fertility intentions in Austria in response to the COVID-19 pandemic. Similarly, Zimmerman et al. (2022) found the pandemic unrelated to major shifts in fertility desires in Kenya. Highly relevant to the context of this study, Svallfors (2022) examined the association between area-level armed conflict and fertility preferences in Colombia and found no differences in fertility desires between women living in areas affected by violent unrest and women living in unaffected areas. However, given the cross-sectional design of the study, Svallfors (2022)’s results are subject to confounding and cannot reveal potential underlying mechanisms.

As in the case of realized fertility, predictions about how exposure to a violent environment affects fertility desires are ambiguous, with potentially heterogeneous effects across groups. In microeconomic theory, higher educational attainment increases the “opportunity costs” of childbearing, such that violence may predominantly affect higher-educated women (Schmidt, 2008; Adserà & Menendez, 2011). Higher-educated women may be better informed about the contextual level of violence and therefore respond by adjusting their fertility desires. Homicide exposure may also affect fertility desires differently across parity levels and age groups. Research suggests that childless women and women in their 20s and 30s may exhibit stronger behavioral fertility response to mortality shocks (Nobles et al., 2015). Changes in fertility desires among reproductive-age women upon the Mexican “Drug War” have not been previously studied. The second objective of this study is therefore to analyze the association between changes in area-level exposure to homicides and changes in fertility desires among Mexican women aged 15–45 between 2002 and 2012.

Given the lack of evidence on the Mexican context, we advance two competing hypotheses. First, we know that risk aversion and uncertainty increased in the Mexican context due to violence (Brown et al., 2019; Villarreal & Yu, 2019), which predicts a negative effect. Second, the literature predicts that any effect would be larger among higher-educated and childless women. Therefore, we hypothesize that:

#### H2a

Rising homicides are associated with decreased fertility desires in Mexico, and the association is larger for higher-educated and childless women.

As discussed above, the empirical results by Svallfors (2021) suggest “remarkable stability” in fertility desires in the wake of prolonged armed conflict in Colombia. As such, we hypothesize that:

#### H2b

Overall, as well as across groups, rising homicides are not associated with fertility desires in Mexico.

## Data and Methods

Our analysis consists of two parts. The first examined the association between homicides and fertility rates at the municipality level; the second used survey data at the individual level to study the association between municipal-level homicides and fertility desires. The municipal-level homicide rates, used in both parts of the analysis, were calculated for 2443 municipalities^1^ across 32 states using vital statistics published by the National Institute of Statistics and Geography (*Instituto Nacional de Estadística*,* Geografía e Informática*, INEGI) and data from the Census of Population and Housing conducted by INEGI in 2000, 2010, and 2020. The numerators were obtained using death certificate microdata (INEGI, n.d. a). As part of this work, we have prepared a public repository with the homicide rate data (Gargiulo et al., 2023).

In the first part of the analysis, we linked homicide rates to municipal-level indicators of fertility to study the area-level association between homicides and fertility. Fertility rates were calculated using data from vital statistics published by INEGI, and population counts published by CONAPO in the 2023 *Conciliación Demográfica* (SGCONAPO, 2023). Yearly age-specific municipal-level birth counts for 2000–2020 were calculated using birth certificate microdata (INEGI, n.d. b).

The association between homicides and fertility rates may be confounded by municipality socio-economic characteristics. Thus, we derived socio-economic indicators for each municipality using data from CONAPO’s *Índices de marginación* (Villasana Ocampo et al., 2023). We also calculated state-level unemployment rates using data from the *Encuesta Nacional de Empleo* (ENE) for 2000–2004 and from the *Encuesta Nacional de Ocupación y Empleo* (ENOE) for 2005–2020. All the area-level variables are summarized in the Online Supplement Section A, Table S1 for years with an available census or population count.

For the second part of the analysis, which concerns fertility desires, we link the municipal-level homicide rates to data from the Mexican Family Life Survey (MxFLS). This is a longitudinal survey representative of the Mexican population in 2002, covering the period before and after the onset of the so-called “Drug War.” The MxFLS collected three waves of data: 2002 (MxFLS1), 2005–2006 (MxFLS2), and 2009–2012 (MxFLS3). The baseline survey has information on approximately 35,600 individuals in 8440 households, and covers 150 municipalities across 16 states throughout Mexico. We restricted our target population to women of reproductive age (15–45), for whom fertility desires are relevant. Previous research has shown that survey drop-out in the MxFLS is low among this group of women (around 6%) and unrelated to homicide rates (Brown, 2018). As we are mainly interested in change over time, our analytic sample consisted of 6,341 women observed at least twice in the survey. The total number of observations is 13,646. For each observation, we linked the municipal homicide rate based on month of interview and municipality of residence. The MxFLS contains data on various aspects of family life, including fertility desires for women of reproductive age. The MxFLS is appropriate for the purposes of our study because data collection was conducted some time before (2002), immediately before (2005–2006), and after (2009–2012) the rise in the homicide rate. The data collection period for wave 3 (2009–2012) included some of the most violent years on record in Mexico (Espinal-Enríquez & Larralde, 2015).

### Measures

#### Municipal-Level Outcome: Fertility Rates

We measured municipal-level fertility using three indicators: the TFR, the CBR, and birth counts. We consider these indicators as complementary: the TFR is age-standardized, thus comparable across municipalities and over time; the CBR is a crude rate, which is better aligned with the homicide rate but does not take into account the population age structure. Finally, unlike rates, birth counts are not calculated from population size, which may change in response to violence. We report the results from the TFR analysis in the paper, but analyses using the CBR and birth counts are available in Online Supplement Sections B and C.

Births were assigned to the municipality where the mother was residing at the time of birth and grouped into 5-year age groups based on the mother’s age when the birth occurred. Births where the mother’s municipality of residence or age at birth were missing were excluded from these counts. We also excluded births where the mother was not residing in Mexico at the time of the birth. Together, these criteria excluded fewer than 1% of all birth certificate data available during the study period. To calculate TFR, age-specific fertility rates were calculated using age-specific municipality-level birth counts and age-specific municipality-level female population counts. The CBR was calculated by dividing the number of births with the minimum required information (municipality of residence and mother’s age at birth) by the total population counts for each municipality-year from 2000 to 2020.

#### Individual-Level Outcome: Fertility Desires

Our outcome of interest in the second part of the analysis was the total desired fertility, measured by the sum of current parity and additional fertility desires. We generated this variable from two survey questions in the MxFLS. The first asked women of reproductive age: “How many (more) children would you like to have?” at each wave. The values of this variable ranged from 0 (no more children) to 12. The second asked women to report any children ever born (at wave 1) or that were born since the previous wave (at waves 2 and 3), which ranged from 0 to 13. For each wave, we set our outcome as the sum of these two numbers. This likely provides an overestimation of fertility desires, as it assumes that all parities were desired. However, for the scope of this analysis, we were interested in changes in fertility desires over time, rather than desired family size. Accounting for the number of children born between waves is essential to isolate changes in desires from changes in parity that get women closer to their desired fertility. For example, consider a woman who reports desiring two additional children at wave 1, has one child between waves 1 and 2, and reports desiring one additional child at wave 2. The total desired fertility for this woman remains stable at two children, and the change in her fertility desires from the previous wave is zero.

#### Municipal-Level Homicide Rates

Our independent variable of interest in both of our analyses was the homicide rate at the municipal level. Deaths were classified as homicides if they were assigned causes of death X95–Y09 according to the 10th Revision of the International Classification of Diseases (WHO, 2004). Of death certificates with these causes of death listed, records missing information about the year and month when the death occurred, as well as the state and municipality where the death occurred were excluded. This information was missing in less than 0.1% of records of homicide deaths. Records of homicides that occurred outside of Mexico were also excluded. The numerator of the homicide rates was then calculated as the total number of homicide deaths containing the minimum required information (year, month, municipality, and state of death) for each municipality-month from January 2000 through December 2020. Mid-year municipality-level population counts were calculated by linearly interpolating municipality-level population counts between census years. Municipal-level monthly crude homicide rates were then calculated by dividing the monthly homicide counts by the monthly population exposure and multiplying by 100,000.

#### Municipal-Level Socio-Economic Controls

Socio-economic indicators at the municipality level included the percentage of the population aged 15 and older who are illiterate; the percentage living without electricity; the percentage living in overcrowded households; and the percentage of people living with less than two minimum salaries. As these indicators are only available for years when a census or population count was conducted (every five years), we linearly interpolated missing values between years. The unemployment rate at the state level was calculated as the average of the number of unemployed individuals (reported quarterly) divided by the economically active population aged 15–64 years.

#### Individual-Level Moderators and Controls

To test for heterogeneity in the association between homicide rates and fertility desires, we used an indicator for educational attainment, categorized as “less than secondary schooling,” “secondary schooling,” “high school,” and “college or higher.” We also defined current parity based on the number of children the woman currently has, which we recode das a binary indicator of whether each woman has any children or not. Finally, we tested for whether the associations differed by age group, recoding the age variables into four categories: 15–19, 20–29, 30–49, and 40–45 years. Based on the above literature on fertility preferences (Buber-Ennser et al., 2024; Nobles et al., 2015; Svallfors, 2021) we included control variables for women’s age in years (as an interval level variable), partnership status (whether married/cohabiting or not), employment status (not working; working less than 30 h per week; working 30 or more hours per week); equivalized household consumption (in 1000s of pesos; for the equivalence scale, see Hagenaars et al., 1994); household size; and self-rated health (“poor,” “fine,” “good,” or “very good”). Additional variables for fear of victimization are available in the MxFLS. Fear of victimization may be a potential mechanism for a reduction in desired fertility as a consequence of homicidal violence. As no evidence of such mediation emerged from the models, however, indicators for victimization were excluded from the analysis.

### Analytic Approach

Our first objective was to study the association between the homicide rate and fertility rates at the municipality level between 2000 and 2020 across 2,443 Mexican municipalities. We started with a linear model for the TFR based on the first or second lag of homicides with municipality fixed-effects (${\gamma}_{m}$):

$${TFR}_{mt}={\beta}_{1}{homicide.rate}_{m,t-n}+{\boldsymbol{\gamma}}_{\boldsymbol{m}}+{\varepsilon}_{mt}$$

The coefficient gives the association between a one-unit increase in the homicide rate in the previous period and the current TFR. The municipality fixed-effects ${\boldsymbol{\gamma}}_{\boldsymbol{m}}$ account for differences in time-invariant municipality characteristics that may affect fertility and homicides, and allow us to isolate the average association between homicides and fertility (${\beta}_{1}$) within municipalities. We alternatively plug the one-year and two-year lag of the homicide rate into the model, as associations may be delayed due to conception and gestation periods. The unadjusted association is likely to be confounded by municipality characteristics linked with homicides and fertility. Socio-economically disadvantaged municipalities may have higher TFR and be more likely to experience rising homicides. We therefore controlled for the municipality socio-economic indicators summarized in Online Supplement Table S1. The subsequent equation is as follows, where ${\boldsymbol{X}}_{\boldsymbol{m}\boldsymbol{t}}$ indicates a vector of time-varying municipality-level covariates:

$${TFR}_{mt}={\beta}_{1}{homicide.rate}_{m,t-n}+{\boldsymbol{\beta}}_{\boldsymbol{i}}{\boldsymbol{X}}_{\boldsymbol{m}\boldsymbol{t}}+{\gamma}_{m}+{\varepsilon}_{mt}$$

We repeated the same analysis for the CBR, comparing results from fixed-effects models with and without controls for municipal-level characteristics. For birth counts, we fit fixed-effects Poisson regressions controlling for population size, subsequently adding controls for socio-economic characteristics at the municipality level. Again, these are available in Online Supplement Sections B and C, respectively.

Although we controlled for municipal-level socio-economic changes that may be correlated with both homicides and the TFR, fixed-effects models do not account for the potential presence of time-varying unobserved confounders, nor for heterogeneous effects over time. As a complementary analysis, we performed a staggered DID analysis following the approach recently proposed by Callaway and Sant’Anna (2021). This estimates the average treatment effect on the treated (ATT) by comparing trends in the outcome between units that received treatment at different points in time, and never-treated units. This method accounts for unobserved time-varying heterogeneity under the parallel trends assumption. We defined two treatments: an increase in homicides by 20/100,000 population, or higher; and an increase in homicides by 50/100,000 population, or higher, between two and one years prior. We primarily examined absolute rather than relative thresholds for change, due to the nature of the research question and data. First, large absolute spikes in homicide are likely to have large effects in both high-violence and low-violence municipalities, while the effect of a percentage increase from very low levels may be substantially small. Second, the majority of municipalities experienced an increase in homicides by 100% or more over the period considered. With these potential limitations in mind, in Online Supplement Section D we present results from using two relative thresholds: an increase in the homicide rate by 100% or more, and an increase in the homicide rate by 500% or between two years and one year prior.

We compared municipalities that experience such increases in homicides at any point between 2007 and 2020 with municipalities that never experience them. The assumption is that, once the municipality experiences a spike, it remains affected until the end of the observation period. An increase in the homicide rate by 20/100,000 or higher between one year and the next is detected in around 10% of observations (municipality-years). Among the 2,443 municipalities for which we have data, 1669 (67.65%) experienced an increase in the homicide rate of this magnitude at least once between 2007 and 2020. Increases by 50/100,000 or more were observed in around 4% of all observations: 847 municipalities (34.33%) experienced such an increase at least once between 2006 and 2020. These represent considerable spikes, given that the overall homicide rate for the Americas in 2017 was 17/100,000 (UNODC, 2019). These figures give an idea of the intensity and territorial spread of homicidal violence in Mexico during this period.

In the staggered DID approach, treated units are grouped into $g$ groups based on the time $t$ at which they are first exposed to treatment. One ATT per treatment group is estimated from:

$$ATT(g,t)=\left[E\left(Y\right)(g{)}_{t}-E(Y\left)\right(NT{)}_{t}\right]-\left[E\left(Y\right)(g{)}_{t-1}-E(Y\left)\right(NT{)}_{t-1}\right]$$

where E(Y) indicates the expected value of the outcome, TFR, and NT indicates the “never treated” group. The estimator takes the difference between the difference in average outcomes between treated and never-treated units at time $t$ and $t-1$. Just like standard DID designs, it assumes parallel trends. In our case, this means that in the absence of a spike in homicides between $t-2$ and $t-1$, municipalities affected by a homicide spike would have experienced the same change in TFR as municipalities that never experienced such a spike. For this to hold, never-treated municipalities should be as comparable as possible to those affected. Therefore, we clustered municipalities by state, and report results with and without controls for the socio-economic characteristics described above (see Online Supplement Table S1), all measured in 2005, as post-treatment covariates are potentially affected by treatment (Callaway & Sant’Anna, 2021). The ATTs for each group are then combined into a single ATT following Callaway and Sant’Anna (2021). In Online Supplement Section B, we replicated all the above analyses using the CBR as a measure of municipal-level fertility.

Our second objective was to assess how changes in the municipality homicide rate over time correspond to changes in the desired number of children for women of reproductive age (15–45).^2^ Our statistical analysis relied on linear models of the change in total desired fertility since the previous wave, net of any children born between waves. We fit the following linear regression model:

$${fert.desires}_{imt}={\beta}_{1}{homicide.rate}_{m,t-n}+{\boldsymbol{\beta}}_{\boldsymbol{i}}{\boldsymbol{X}}_{\boldsymbol{i}\boldsymbol{m}\boldsymbol{t}}+{\theta}_{i}+t\cdot{\gamma}_{m}+{\varepsilon}_{imt}$$

Each woman $i$ lives in municipality $m$ at month $t$. We regressed changes in fertility desires on the homicide rate in the woman’s municipality of residence, using alternative lags of three (*n* = 3) and six months (*n* = 6). These have been identified as the lower- and upper-bound of the relevant period during which a spike in homicide violence may affect fertility desires (Weitzman et al., 2021). We controlled for women’s characteristics associated with fertility desires expressed by the vector ${\boldsymbol{X}}_{\boldsymbol{i}\boldsymbol{m}\boldsymbol{t}}$, which included age in years, marital status, educational attainment, work status, number of people in the household, household consumption, and the woman’s self-rated health. ${\theta}_{i}$ indicates woman-specific intercepts, while the term $t\cdot{\gamma}_{m}$ represents a municipality time trend, accounting for changes in fertility desires within municipalities over time that are unrelated to homicides. Finally, ${\varepsilon}_{imt}$ indicates the woman-specific error term.

We adopted two alternative specifications of the model. The first treats ${\theta}_{i}$ as woman-specific random intercepts, resulting in a random-effects model with observations nested within women. The model accounts for the clustering of observations within individuals, and ${\beta}_{1}$ summarizes co-variation in the homicides and fertility desires both across-women as well as within-women over time. As a second specification, we treat ${\theta}_{i}$ as women-specific fixed-effects. This model only relies on variation within women over time to estimate ${\beta}_{1}$ and other coefficients, thus accounting for unobserved time-invariant differences in fertility desires between women.

A potential concern with estimating the effect of an increase in the municipal homicide rate on fertility desires is selective out-migration of women from the most affected municipalities, which may be related to characteristics that independently influence fertility desires (such as the family’s socio-economic status). To minimize the risk of bias from selective migration, we followed Brown (2018) in fixing the municipality of residence at wave 2 (2005–06) for women who migrate. Given that the “Drug War” began in December 2006, it is unlikely that women were able to anticipate the increase in homicides at wave 2 of the MxFLS.

## Results

### Changes in TFR and Homicide Rate Across Mexican States, 2000–2020

**Fig. 2:**
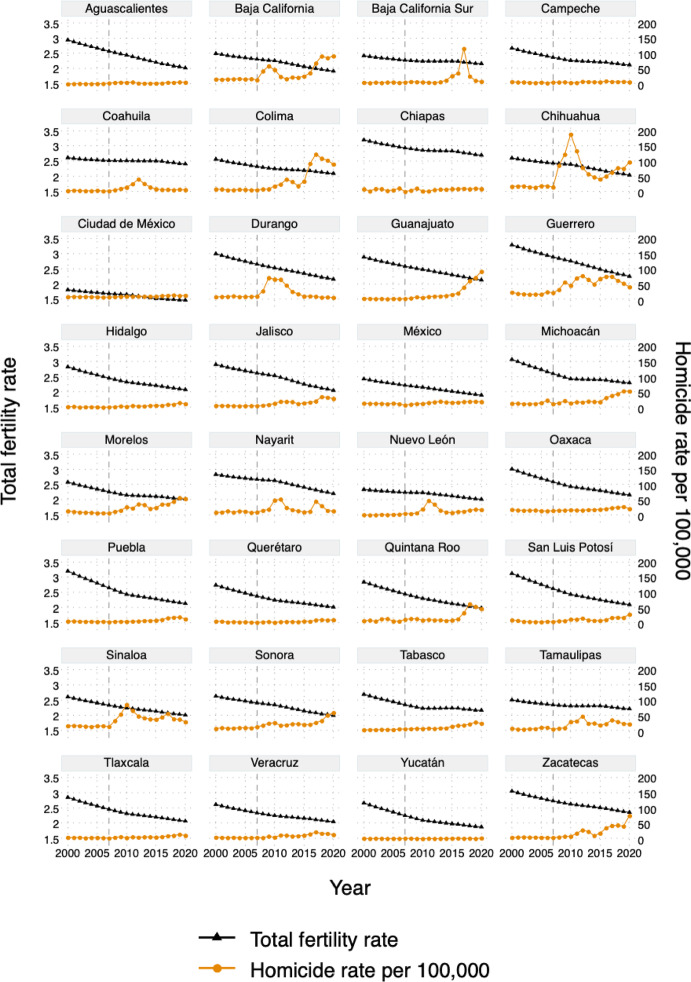
Trends in total fertility rate and homicide rate per 100,000 inhabitants across Mexican states (2000–2020), with reference line at 2007

We begin by providing an overview of changes in TFR and the homicide rate for each of the 32 Mexican states. Figure 2 summarizes trends for 2000–2020, with TFR on the left-hand side scale and the homicide rate per 100,000 people on the right-hand side. The decline in the TFR appears linear over time, with states with higher TFR in 2000 (e.g., Guerrero, Michoacán, Puebla, and San Luis Potosí) experiencing steeper declines between 2000 and 2010. Overall, the TFR declined from around three children per woman in 2000 to around two in 2020, with the exception of Mexico City, where it was consistently below two children per woman and declining throughout the period. In some states (e.g., Campeche, Chiapas, Puebla, Tlaxcala), an initially more rapid fertility decline (2000–2010) was followed by slower reductions (2010–2020). However, from visual inspection, the change in slopes does not seem to be systematically related to trends in the homicide rate. The figure shows the heterogeneous effect of the “Drug War” across the country. Some states (e.g., Chihuahua, Durango, Sinaloa) experienced large spikes in homicide rate in the initial years of the “Drug War” (up to 180/100,000 in 2010 in Chihuahua). Other states were mainly affected after 2015 (e.g., Baja California Sur, Colima, Guanajuato). Others remained relatively unaffected (e.g., Aguascalientes, Yucatán). At the state level, the Spearman correlation coefficient between the TFR and the homicide rate is − 0.29, indicating a weak negative correlation. This may be attributable to ongoing fertility decline during a period when homicides substantially increased, and more granular data are needed to test for this association. A similar picture emerges for the CBR in Online Supplement Figure S1.

### The Association Between Homicides and Fertility, 2000–2020

To study the association between homicides and fertility across municipalities, we begin by modelling the TFR on the homicide rate for 2443 municipalities over time. Table 1 shows the coefficients from municipality fixed-effects models with and without controls for municipality deprivation and state unemployment. The first and second lags of the homicide rate are studied in alternative models, with the other coefficients remaining virtually unchanged. Regardless of which lag is considered, we find a small negative association between an increase in homicides and the TFR within municipalities over time in the unadjusted model. Specifically, an increase in homicide rates by 10/100,000 is associated with a reduction in TFR between 0.02 and 0.03 children per woman. However, when time-varying controls for municipality deprivation indicators are included, the association between TFR and the homicide rate goes to zero in both cases. An alternative model specification with first differences also shows no association, whether or not socio-economic controls are included, with coefficients presented in Online Supplement Section A, Table S2. When analyses are repeated using the CBR in Online Supplement Section B, a similar picture emerges. In the fixed-effects models (Table S3), the negative association gets very close to zero once municipal-level controls are added, with a homicide rate increase by 10/100,000 associated with a decline in the CBR by 0.2–0.3/1000 children; in the first-difference models (Table S4), no association is found. In addition, when fitting fixed-effects Poisson regression models for birth counts on homicide counts (instead of rates) in Online Supplement Section C (Table S5), we obtain a null result. Although the association between homicides and fertility is slightly negative, the very small coefficients do not provide robust evidence for our hypothesis H1 of a negative association between violence and TFR in Mexico.

**Table 1:** Coefficients and standard errors from fixed-effects models for total fertility rate across municipality-years

	No controls	With deprivation controls
Homicide rate, 3-month lag	− 0.003 (0.000)***	− 0.000 (0.000)
Homicide rate, 6-month lag ^a^	− 0.002 (0.000)***	− 0.000 (0.000)
% Illiterate		0.065 (0.002)***
% with no electricity		0.021 (0.001)***
% in overcrowded HHs		0.026 (0.001)***
% Communities < 5,000 pop.		− 0.002 (0.001)**
% < 2 minimum salaries		0.000 (0.000)
% Unemployed (state)		0.001 (0.003)
*N* (municipalities)	2443	2443
*N* (observations)	34,144	34,144
Intraclass correlation coefficient	0.45	0.69

HH indicates “household;” All models include municipality fixed-effects

^a^Coefficients from the same model, where the 2nd lag of homicide rate substitutes the 1st lag

^†^ *p* < .10, *p < .05, **p < .01, ***p < .001

**Fig. 3:**
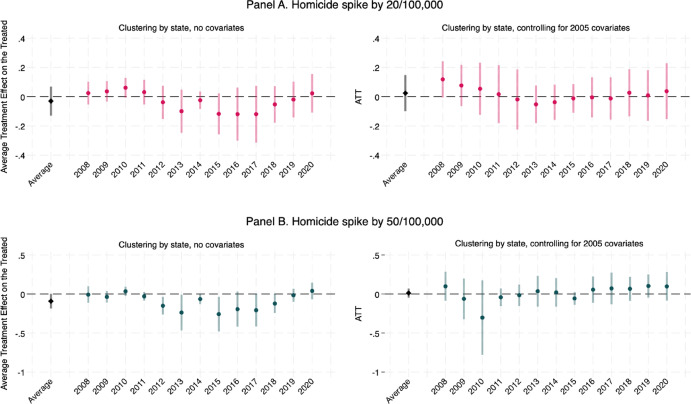
Average treatment effect on the treated of increases in the homicide rate by 20 and 50 per 100,000 in the previous year, by year of exposure

As a complementary approach, we use a staggered DID design (Callaway & Sant’Anna, 2021). Figure 3 displays the estimated ATTs for each group defined by exposure to a spike in homicides by either 20/100,000 or more (Panel A) or 50/100,000 or more (Panel B) between two and one years prior, with and without controls for municipality-level deprivation. In Online Supplement Section D, we use the same approach but define the thresholds in a relative sense, as a 100% increase and a 500% increase in the homicide rate between two years and one year prior.

In all cases, we find no effect of a homicide spike on TFR for the entire period considered, again providing no evidence for hypothesis H1. The aggregated ATTs for a homicide spike by 20/100,000 (with 95% confidence intervals) are − 0.049 [− 0.164, + 0.065] without municipality deprivation controls, and − 0.000 [− 0.148, + 0.148] with controls. The aggregated ATTs for a spike by 50/100,000 are − 0.101 [− 0.225, + 0.024] without controls, and + 0.042 [− 0.053, 0.138] with controls. When the analyses are repeated for the CBR, a similar picture emerges with no effect of homicide spikes on fertility (Fig. 2). Furthermore, when repeating the analyses for two relative thresholds—that is, increases in the homicide rate by 100% and 500% between two years and one year prior—we find no overall effect of an increase in the homicide rate on either the TFR (Online Supplement Figure S3) or the CBR (Online Supplement Figure S4). When homicides increase by five times the original rate (that is, a 500% increase), we find a small negative effect on TFR and CBR in the years between 2013 and 2016, by 0.1 children per woman for the TFR and less than 1/1,000 births for the CBR. These changes are negligible, given the extent of such change.

### Homicide Rates and Changes in Fertility Desires, 2002–2012

**Fig. 4:**
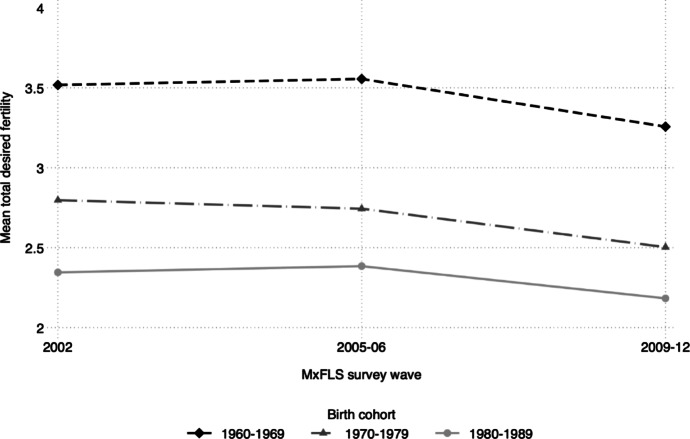
Change in mean total desired and achieved fertility among women aged 15–45 years in the MxFLS, by birth cohort

Moving on to fertility desires, Table 2 displays and compares unweighted sample characteristics of women at each wave of the MxFLS. Total desired fertility decreases over time and especially between waves 2 and 3, which is partly the result of falling desired fertility, and partly deriving from the fact that higher-parity women leave our analytic sample as they reach age 45. To complement these numbers, Fig. 4 displays changes in fertility desires net of woman-specific births for the overall sample and across cohorts born in the 1960, 1970, and 1980. Once the birth of additional children between survey waves is accounted for, fertility desires are relatively stable over the 10-year period, with some decline between 2005 and 2009 (by around 0.2 children per woman). There are large differences across cohorts, as women born in the 1980’s desire one fewer child on average than women born in the 1960's, in line with the trends in TFR decline discussed above (see Fig. 2). A substantial proportion of sampled women complete university education during the survey period (from 9.13 to 15.60%), and there is an increase in the proportion of women working full-time (from 22.62 to 30.28%).

**Table 2:** MxFLS analytic sample characteristics by survey wave

	Wave 1 (2002)	Wave 2 (2005–2006)	Wave 3 (2009–2012)	Test for differences across waves^a^
Total desired fertility (mean)	2.969	2.954	2.554	***
Additional children desired (mean)	1.114	0.961	0.861	***
Number of children (mean)	1.855	1.993	1.693	***
Age in years (mean)	27.990	31.636	32.444	***
Educational attainment				
None (%)	38.340	38.320	32.630	***
Secondary (%)	35.420	33.310	34.360	
High School (%)	17.110	14.980	17.400	
University (%)	9.130	13.390	15.600	
Married or cohabiting (%)	60.600	62.790	63.160	*
Work status				
Not working (%)	66.770	64.180	58.620	***
Working < 30 h/week (%)	10.600	10.620	11.100	
Working ≥ 30 h/week (%)	22.620	25.190	30.280	
HH expenditure in 1000s pesos (mean)	1.600	1.592	1.981	**
HH size (mean)	5.175	5.321	5.708	***
Self-rated health				
Poor (%)	3.130	2.660	3.070	***
Fine (%)	42.290	39.650	41.800	
Good (%)	49.700	50.190	47.990	
Very good (%)	4.870	7.510	7.140	

*N* = 6341 women aged 15–45 years; Sample statistics are unweighted; HH indicates “Household.”

^a^One-way ANOVA or a chi-square test of differences in the values of the variable across waves

^†^*p* < .10, *p < .05, **p < .01, ***p < .001

Did changes in the municipal-level homicide rate correspond to changes in women’s desires for (additional) children? Table 3 displays and compares the coefficients from random-effects and fixed-effects models for fertility desires. The main coefficients of interest are the three-month and six-month lag of the homicide rate, which are plugged into separate models but reported here together, since the other coefficients remain unchanged when switching between the two. The results from both random-intercept and fixed-effect models show no change in fertility desires subsequent to increases in exposure to homicides. Coefficients on the municipality homicide rate are close to zero and not statistically significant. From the random-intercept model, we observe that older women, married women, and women living in larger households have higher average desired fertility, while higher-educated women and women working full-time have lower average desired fertility. These differences disappear once we isolate changes within women over time. In the fixed-effects model, completing university is associated with an increase in fertility desires, and having an additional household member is correlated with lower desired fertility. Online Supplement Section E, Table S6 reports results from the models in Table 3, but excluding municipality time trends for comparison. Even then, no overall association between homicide rates and fertility desires is detected.

**Table 3:** Coefficients and standard errors from random intercept and fixed-effects models for fertility desires among women aged 15–45 years

	Random intercept model	Fixed-effects model
Homicide rate, 3-month lag	− 0.000 (0.000)	− 0.000 (0.001)
Homicide rate, 6-month lag^a^	− 0.001 (0.000)	− 0.000 (0.000)
Age (years)	0.055 (0.002)***	− 0.007 (0.012)
Education (ref=none)		
Secondary	− 0.263 (0.033)***	0.033 (0.057)
High school	− 0.239 (0.042)***	0.137 (0.073)
University	− 0.250 (0.047)***	0.219 (0.086)**
Married or cohabiting	0.251 (0.028)***	0.056 (0.039)
Work status (ref = not working)		
< 30 h/week	− 0.034 (0.032)	0.033 (0.031)
≥ 30 h/week	− 0.089 (0.026)***	0.018 (0.037)
HH consumption	0.000 (0.001)	0.000 (0.001)
HH size	0.093 (0.006)***	− 0.045 (0.011)***
Self-rated health: poor		
Fine	− 0.035 (0.057)	− 0.033 (0.064)
Good	− 0.038 (0.058)	− 0.018 (0.065)
Very good	0.073 (0.068)	0.093 (0.077)
Municipality × time	Yes	Yes
*N* (women)	6341	6341
*N* (observations)	13,646	13,646
Intraclass correlation coefficient	0.573	0.792

HH indicates “household;” models include municipal-level time trends

^a^Coefficients from the same model, where the 2nd lag of homicide rate substitutes the 1st lag

^†^*p* < .10, *p < .05, **p < .01, ***p < .001

The null result may mask heterogeneities in the association between homicide rates and fertility desires. Panel A of Table 4 reports coefficients for the municipal homicide rate from the same models, fitted separately by the highest level of educational attainment attained during the survey period (2002–2012). Panel B reports separate results for women with and without children based on parity at wave 2, right before the increase in homicides. Panel C shows results by age group. The unabridged tables corresponding to Table 4 can be found in Online Supplement Section F (Tables S8–S13). These results are consistent with there being no association between homicide rates and fertility desires. All the coefficients are close to zero, and none are statistically significant except a very small negative association between the 3-month lag of the homicide rate and fertility desires among women aged 40–45. Online Supplement Table S7 shows the same results from models without municipality time trends. These show some small negative associations between homicide rates and fertility for lower and secondary-educated women, women with children, and above age 20, which are only detected in the random-effects models, and mostly disappear once individual fixed-effects are added. This suggests that any small negative association may be driven primarily by unobserved differences across women in fertility desires that are also correlated with homicides exposure at the municipality level. Overall, we find no evidence for hypothesis H2a of a negative association and, in line with H2b, we conclude that there is no association between homicide rates and average desired fertility in Mexico.

**Table 4:** Coefficients and standard errors for the 3-month and 6-month lag of the homicide rate from random-effects and fixed-effects models for fertility desires

	Random intercept model		Fixed effect model	
	Homicide rate lag:		Homicide rate lag:	
	3-month	6-month	3-month	6-month
*Panel A. By education*				
No formal education (*N =* 2008, *n =* 4452)	− 0.001 (0.001)	0.000 (0.001)	− 0.001 (0.001)	− 0.000 (0.001)
Secondary (*N =* 2036, *n =* 4481)	− 0.000 (0.001)	− 0.001 (0.001)	0.000 (0.001)	− 0.001 (0.001)
High school (*N =* 1196, *n =* 2437)	0.001 (0.001)	0.000 (0.001)	0.000 (0.002)	− 0.001 (0.001)
University (*N =* 1097, *n =* 2273)	− 0.000 (0.001)	0.000 (0.001)	0.000 (0.001)	0.000 (0.001)
*Panel B. By parity*				
No children (*N =* 2184, *n =* 4109)	− 0.000 (0.001)	0.000 (0.001)	0.000 (0.001)	− 0.000 (0.001)
Has children (*N =* 4153, *n =* 9534)	− 0.000 (0.001)	− 0.000 (0.000)	− 0.000 (0.001)	− 0.001 (0.001)
*Panel C. By age group*				
15–19 years (*N =* 1164, *n =* 1446)	0.000 (0.002)	0.001 (0.002)	− 0.008 (0.012)	0.007 (0.011)
20–29 years (*N =* 3289, *n =* 4704)	− 0.000 (0.001)	0.000 (0.001)	− 0.001 (0.001)	− 0.002 (0.001)
30–39 years (*N =* 3219, *n =* 5138)	0.001 (0.001)	− 0.000 (0.001)	0.001 (0.001)	− 0.001 (0.001)
40–45 years (*N =* 1756, *n =* 2354)	− 0.003 (0.002)*	− 0.001 (0.001)	− 0.004 (0.003)	0.001 (0.003)

Separate models fitted by educational attainment, parity, and age group; All models control for covariates and municipal-level time trends; *N* is number of women, *n* is number of observations. Unabridged tables can be found in Online Supplement Section F (Tables S8–S13)

^†^*p* < .10, *p < .05, **p < .01, ***p < .001

## Discussion and Conclusion

Our study has comprehensively investigated the fertility response to the increase in homicides in Mexico over the past 20 years (Dell, 2015). We find that, at the municipality level, fertility rates have very small to no marginal associations with homicide rates once socio-economic characteristics are taken into account. However, homicidal violence during the “Drug War” has been unequally distributed, with different areas affected to widely different extents at various points in time (Espinal-Enríquez & Larralde, 2015; Rios, 2013). When we model such heterogeneity through a staggered DID approach (Callaway & Sant’Anna, 2021), we find that areas where homicide rates increased by five times or more in a year between 2013 and 2016 experienced slightly faster reductions in fertility, by around 0.1 children per woman based on the TFR. These years of the conflict were among the most violent on record (Espinal-Enríquez & Larralde, 2015). Given how small these effects are, our expectation about a negative fertility response to the “Drug War” was not verified. Such small negative effects may be partly the result of internal migration out of the most violent municipalities (Verwimp et al., 2020).

We dug deeper into whether and how homicidal violence has affected fertility by looking at changes in fertility desires among reproductive-age women living in 150 municipalities between 2002 and 2012, accounting for selective migration out of the most affected areas (Brown, 2018). Results indicate no association between the municipality-level homicide rate and total desired and achieved fertility. Consistent with previous research (Berrington & Pattaro, 2014), we show that while differences in fertility desires exist across women of different socio-economic status, the main predictors of within-woman changes in desired fertility are completing education and changes in household size. We do not find evidence of heterogeneity by education, childlessness, or age group, which are commonly conceptualized moderators (Nobles et al., 2015; Schmidt, 2008), reinforcing our confidence in a true null result. The MxFLS data shows a decline in desired family size across birth cohorts, with women born in the 1980s having a total desired and achieved family size of 2.2 children, compared to 3.3 children for women born in the 1960s. Overall, our results reach the same conclusions as Svallfors’ (2022) indication of “remarkable stability” subsequent to the armed conflict in Colombia. Oure results are similarly in line with recent literature on the stability of fertility desires in the face of COVID-19-related uncertainty (Buber-Ennser et al., 2024; Zimmerman et al., 2022).

Our study has limitations to acknowledge. While homicide rates are commonly used in the literature and considered to be the most complete of the data documenting crime and violence in Mexico (Brown 2018; Caudillo and Lee 2023a), homicide data is still under-reported. In the context of the “Drug War,” the simultaneous rise in disappearances poses an additional barrier to complete homicide documentation using vital statistics. As of May 30, 2025, Mexico’s National Search Commission (*Comisión Nacional de Búsqueda*, CNB) reports that there are over 128,000 open and unresolved missing person and disappearance cases (CNB, n.d.). Many of these unresolved cases are likely to be homicides for which a death certificate has not been issued. Moreover, the true number of victims of disappearances may be higher as not all disappearances are reported to the CNB. Alongside the rise in homicides and disappearances, Mexico has also been experiencing a forensic crisis. As of August 2021, state officials reported a backlog of over 52,000 unidentified cadavers in public cemeteries and other institutions (Brewer, 2022; Movimiento por nuestros desaparecidos en México, 2021). Backlogs may delay or prevent the issuance of homicide death certificates. These challenges indicate that homicide rates are likely underestimated in at least some municipalities, although publicly available information does not allow us to identify which municipalities, nor to estimate the potential degree of underreporting.^3^ Moreover, due to varying population size across municipalities, very small municipalities may be over-sensitive to spikes in the homicide rate.

With respect to the fertility data, we only analyze short-term changes in fertility. As such, future research may fruitfully investigate the long-term consequences of the “Drug War” once more data is available. We are also unable to look at changes in related outcomes such as induced abortions, due to lack of data and the fact that abortion was largely illegal—and extremely difficult to measure—for the whole period under study. In our analysis of fertility rates, we cannot account for internal migration out of the most heavily affected areas due to lack of available data. In our analysis of fertility desires, the attrition rate may partly be due to violence-induced migration, but fixing the municipality of residence at its 2005/2006 value for women who migrate does not change the results. When studying fertility desires, we refrain from making causal claims as the longitudinal associations between homicide rates and desired fertility may be affected by unobserved differences between and within women over time. The limited number of municipalities covered in the MxFLS (150 in total) does not allow for a staggered DID approach. The complete lack of association in our data—with all coefficients on the homicide rate close to zero—is indicative of no average change in desires corresponding to increases in homicides, but there may be uncaptured effects in the most heavily affected areas. As mentioned above, we do not have reliable information on fertility intentions, which would help provide a more complete picture of fertility change (Miller, 2011).

The rapid fertility decline observed in Mexico between the 1960s and 2000s was driven by the significant rise in effective contraceptive use and the diffusion of so-called “stopping and spacing” behavior propelled by family planning policies, economic development, urbanization, and educational expansion (Tuiran et al., 2002). Over this period, age at first birth remained low, in line with Latin American patterns of early family formation and smaller parities (Esteve et al., 2022). During the period of the so-called Drug War, Mexico’s fertility has continued to decline, albeit at a slower pace, driven primarily by the decline in first births among women younger than 25, suggesting a postponement of fertility (Pardo et al., 2025). This is again consistent with patterns in other Latin American countries such as Colombia, Chile, and Brazil (Pardo et al., 2025). Explanations for such trends in the region include increases in the use of long-acting contraceptive methods, ideational changes (such as the decline in fertility desires across cohorts observed in our data, see Fig. 4), and structural transformations including expanded educational participation among adolescents and young adults (Pardo et al., 2025).

The relative stability in fertility decline evidenced in our study in no way indicates that the “Drug War” has had no significant effects on the Mexican population. On the contrary, it is a testament of remarkable continuity and resilience in the face of extremely adverse conditions. It is important for future research to continue monitoring trends in sub-national fertility, and to understand the extent to which any potential mismatch between desires and realizations is due to postponement, cohort replacement, or systemic failures in enabling women to achieve their desired family size. If the latter were to be the case, national and local policies should be implemented to improve access to childbearing and child-rearing, from healthcare coverage and insurance to childcare service provision.

## Supplementary Information

Below is the link to the electronic supplementary material.

Supplementary Material 1

## Data Availability

All data used in the analysis are publicly available. Death certificate microdata, used in the calculation of the homicide rates, is available from: https://www.inegi.org.mx/programas/edr/. Censuses and population counts, used in the calculation of the homicide rates, are available from: https://www.inegi.org.mx/programas/ccpv. As part of the project, we created a repository with the homicide rates (10.17605/OSF.IO/U8DC3). Birth certificate microdata, used to calculate the fertility indicators, is available from: https://www.inegi.org.mx/programas/natalidad/. The 2023 *Conciliación Demográfica*, used for population exposures, is available from: https://www.gob.mx/conapo/acciones-y-programas/conciliacion-demografica-de-1950-a-2019-y-proyecciones-de-la-poblacion-de-mexico-y-de-las-entidades-federativas-2020-a-2070. Data from the *Índices de marginación*, used for socioeconomic control variables, is available from https://www.gob.mx/conapo/documentos/indices-de-marginacion-1990-2010 and https://www.gob.mx/conapo/documentos/indices-de-marginacion-2020-284372. Data from the *Encuesta Nacional de Empleo*, used to calculate state-level unemployment rates prior to 2005, is available from: https://www.inegi.org.mx/programas/ene/2004/. Data from the *Encuesta Nacional de Ocupación y Empleo*, used to calculate state-level unemployment rates from 2005, is available from: https://www.inegi.org.mx/programas/enoe/14ymas/. Data from the Mexican Family Life Survey is available from: https://www.ennvih-mxfls.org/english/index.html. Replication code is available from 10.17605/OSF.IO/7F6GQ. Competing interests statement: There are no conflicts of interest or competing interests in this work.

## Footnotes

1 Due to administrative changes, the number of municipalities has varied over time.

2 Results are not sensitive to using 15–49 as the age range.

3 Omission, impunity, and outright collusion on the part of the State also impact the documentation of violence, which may result in under-reporting. See for example, the State’s involvement in the disappearance of 43 students from the rural teachers’ college Raul Isidro Burgos in Ayotzinapa, Guerrero (e.g., Grupo Interdisciplinario de Expertos Independientes, 2023).
