# Supplementary material for: Changes in Fertility Trends and Women’s Fertility Desires in the Wake of the Homicide Surge in Mexico
Source: Popul Res Policy Rev. 2026 Mar 16;45(2):17. doi: 10.1007/s11113-026-09999-x (PMC12992446; doi:10.1007/s11113-026-09999-x)
Supplement: Supplementary file 1 — Supplementary Material 1 [file 11113_2026_9999_MOESM1_ESM.docx]

**ONLINE SUPPLEMENT**

**Changes in Fertility Trends and Women’s Fertility Desires in the Wake of the Homicide Surge in Mexico**

***Table of Contents***

[A. Supplementary Tables for Municipal-Level Analyses 2](#_Toc213237102)

[Table S1 2](#_Toc213237103)

[Table S2. 3](#_Toc213237104)

[B. Results Using Crude Birth Rate Instead of Total Fertility Rate 4](#_Toc213237105)

[Table S3. 4](#_Toc213237106)

[Table S4. 5](#_Toc213237107)

[Figure S1. 6](#_Toc213237108)

[Figure S2. 7](#_Toc213237109)

[C. Fixed-Effects Models for Birth and Homicide Counts Instead of Rates 9](#_Toc213237110)

[Table S5. 9](#_Toc213237111)

[D. Results from Staggered Difference-In-Differences Approach with Relative Thresholds for Changes in the Homicide Rate 10](#_Toc213237112)

[Figure S3. 11](#_Toc213237113)

[Figure S4. 12](#_Toc213237114)

[E. Results from Random and Fixed-Effects Models for Fertility Desires without Municipality Time Trends 13](#_Toc213237115)

[Table S6 13](#_Toc213237116)

[Table S7. 14](#_Toc213237117)

[F. Unabridged Tables Corresponding to Manuscript Table 4 15](#_Toc213237118)

[Table S8. 15](#_Toc213237119)

[Table S9. 16](#_Toc213237120)

[Table S10. 17](#_Toc213237121)

[Table S11. 17](#_Toc213237122)

[Table S12. 18](#_Toc213237123)

[Table S13. 20](#_Toc213237124)

#

# ***A. Supplementary Tables for Municipal-Level Analyses***

**Table S1.** Summary statistics for municipality-level indicators for Census or population count years

|  | **2000** | **2005** | **2010** | **2015** | **2020** |
| --- | --- | --- | --- | --- | --- |
| Homicides per 100,000 pop. | 11.69 | 10.48 | 23.21 | 16.94 | 22.63 |
| Total Fertility Rate | 3.68 | 3.07 | 2.75 | 2.21 | 1.84 |
| Crude Birth Rate per 1,000 pop. | 27.18 | 23.41 | 21.72 | 17.53 | 14.49 |
| Birth counts | 1038.00 | 955.00 | 955.00 | 901.00 | 677.00 |
| **Socio-economic deprivation** |  |  |  |  |  |
| % illiterate | 16.20 | 14.76 | 14.06 | 11.75 | 10.16 |
| % with no electricity | 9.62 | 5.33 | 4.04 | 2.21 | 1.50 |
| % in overcrowded HHs | 52.87 | 48.01 | 44.72 | 36.31 | 26.54 |
| % communities < 5000 pop | 65.70 | 64.61 | 71.99 | 71.67 | 69.96 |
| % < 2 minimum salaries | 65.00 | 58.92 | 61.76 | 55.30 | 82.09 |
| **% unemployed (state)** | 1.37 | 3.11 | 4.42 | 3.77 | 3.74 |
|  |  |  |  |  |  |
| *N* (municipalities) | 2,443 | 2,443 | 2,443 | 2,443 | 2,443 |

Notes: HH indicates “household”

^a^ coefficient from an alternative model.

Sources: authors’ calculations from INEGI (2022); SGCONAPO (2023)

**Table S2.** Coefficients and standard errors from first-difference models for the total fertility rate

|  | **No controls** | **With deprivation controls** |
| --- | --- | --- |
| Lag of homicide rate | -0.000 (0.000) | -0.000 (0.000) |
|  |  |  |
| % illiterate |  | 0.025 (0.007) *** |
| % with no electricity |  | 0.016 (0.004) *** |
| % in overcrowded HHs |  | -0.007 (0.002) |
| % communities < 5000 pop |  | 0.002 (0.001) |
| % < 2 minimum salaries |  | 0.003 (0.001) *** |
| % unemployed |  | 0.005 (0.004) |
|  |  |  |
| *N* (municipalities) | 2,443 | 2,443 |
| *N* (observations) | 34,144 | 34,144 |

Notes: HH indicates “household”

^†^ *p* < .10, * *p* < .05, ** *p* < .01, *** *p* < .001

# ***B. Results Using Crude Birth Rate Instead of Total Fertility Rate***

**Table S3.** Coefficients and standard errors from fixed-effects models for crude birth rate per 1,000 population at the municipality level.

|  | **No controls** | **With deprivation controls** |
| --- | --- | --- |
| Homicide rate, 3-month lag | -0.020 (0.001) *** | -0.002 (0.001) * |
| Homicide rate, 6-month lag^a^ | -0.019 (0.001) *** | -0.003 (0.001) *** |
|  |  |  |
| % illiterate |  | 0.231 (0.014) *** |
| % with no electricity |  | 0.073 (0.008) *** |
| % in overcrowded HHs |  | 0.279 (0.005) *** |
| % communities < 5,000 pop. |  | 0.009 (0.004) *** |
| % < 2 minimum salaries |  | -0.007 (0.003) ** |
| % unemployed |  | 0.043 (0.020) * |
|  |  |  |
| *N* (municipalities) | 2,443 | 2,443 |
| *N* (observations) | 34,144 | 34,144 |

Notes: HH indicates “household”

^a^ coefficient from an alternative model.

^†^ *p* < .10, * *p* < .05, ** *p* < .01, *** *p* < .001

**Table S4.** Coefficients and standard errors from first-difference models for crude birth rate per 1,000 population at the municipality level.

|  | **No controls** | **With deprivation controls** |
| --- | --- | --- |
| Homicide rate, 3-month lag | -0.000 (0.001) | -0.001 (0.001) |
|  |  |  |
| % illiterate |  | -0.006 (0.051) |
| % with no electricity |  | 0.056 (0.026) * |
| % in overcrowded HH’s |  | -0.042 (0.026) |
| % communities < 5000 pop |  | 0.020 (0.012) |
| % < 2 minimum salaries |  | 0.020 (0.006) *** |
| % unemployed |  | 0.070 (0.029) * |
|  |  |  |
| *N* (municipalities) | 2,443 | 2,443 |
| *N* (observations) | 34,144 | 34,144 |

Notes: HH indicates “household.”

^†^ *p* < .10, * *p* < .05, ** *p* < .01, *** *p* < .001

**Figure S1.** Trends in crude birth rate and homicide rate per 100,000 inhabitants across Mexican states

**
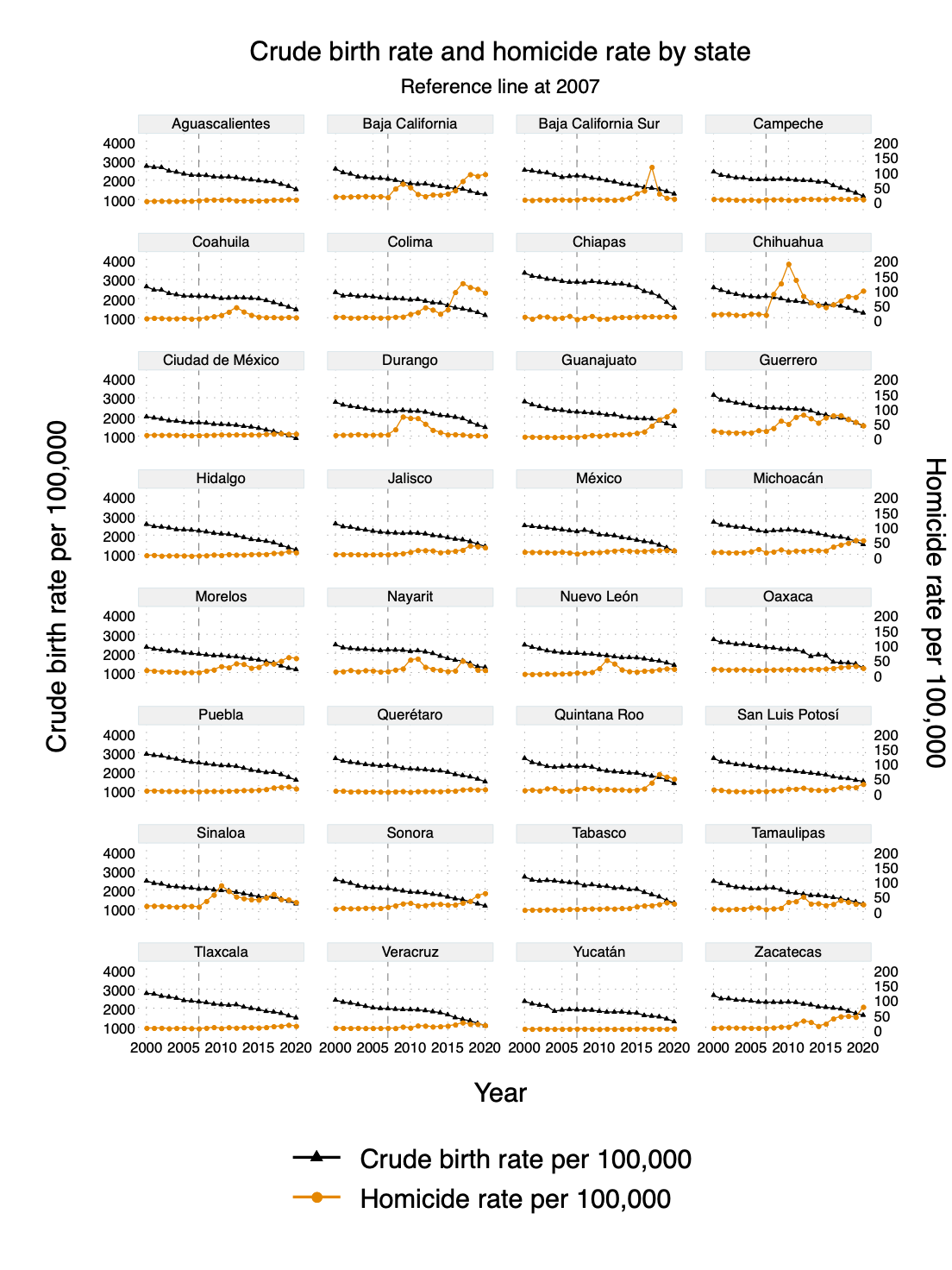
**

**Figure S2.** Average treatment effect on the treated (ATT) of increases in homicide rates by 20/100,000 and 50/100,000 on the crude birth rate, by period of first exposure.


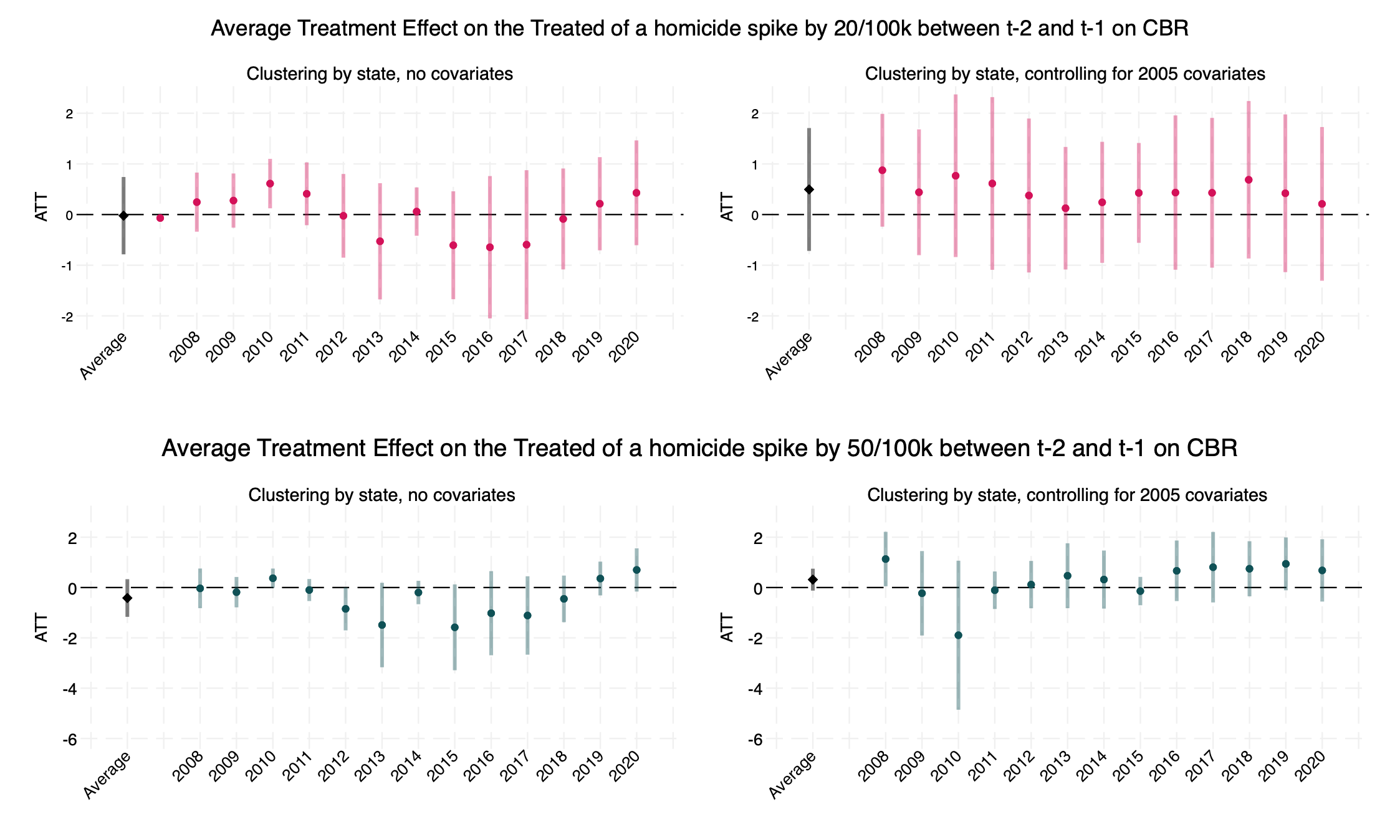


**Overall ATTs with 95% confidence intervals:**

Threshold at 20 homicides, no controls: -0.071 (-0.633 ; 0.774)

Threshold at 20 homicides, with controls: 0.343 (-0.941, +1.626)

Threshold at 50 homicides, no controls: -0.251 (-1.093 ; 0.592)

Threshold at 50 homicides, with controls: 0.441 (-0.169 ; 1.050)

# ***C. Fixed-Effects Models for Birth and Homicide Counts Instead of Rates***

**Table S5.** Incidence rate ratios and standard errors from fixed-effects Poisson regression models for birth counts at the municipality level.

|  | **Control for pop. size only** | **With deprivation controls** |
| --- | --- | --- |
| Homicide Count, 3-month lag | 0.997 (0.001) * | 1.000 (0.000) |
| Homicide Count, 6-month lag^a^ | 0.997 (0.001) * | 0.999 (0.000) * |
|  |  |  |
| Total population | 0.999 (0.000) | 1.000 (0.000) *** |
| % illiterate |  | 0.986 (0.003) *** |
| % with no electricity |  | 1.006 (0.001) *** |
| % in overcrowded HH’s |  | 1.011 (0.001) *** |
| % communities < 5,000 pop. |  | 0.994 (0.002) *** |
| % < 2 minimum salaries |  | 0.994 (0.000) *** |
| % unemployed |  | 0.986 (0.002) *** |
|  |  |  |
| *N* (municipalities) | 2,443 | 2,443 |
| *N* (observations) | 34,144 | 34,144 |

Notes: HH indicates “household.”

^a^ coefficient from an alternative model.

^†^ *p* < .10, * *p* < .05, ** *p* < .01, *** *p* < .001

# ***D. Results from Staggered Difference-In-Differences Approach with Relative Thresholds for Changes in the Homicide Rate***

**Overall ATTs with 95% confidence intervals (Total Fertility Rate)**

Threshold at 100% increase in homicides, no controls: 0.203 (-0.074 ; 0.478)

Threshold at 100% increase in homicides, with controls: 0.021 (-0.256 ; 0.299)

Threshold at 500% increase in homicides, no controls: -0.030 (-0.081 ; 0.019)

Threshold at 500% increase in homicides, with controls: -0.044 (-0.101 ; 0.014)

**Overall ATTs with 95% confidence intervals (Crude Birth Rate):**

Threshold at 100% increase in homicides, no controls: 1.655 (-0.082 ; 3.391)

Threshold at 100% increase in homicides, with controls: 0.358 (-0.949 ; 1.663)

Threshold at 500% increase in homicides, no controls: 0.192 (-0.120 ; 0.505)

Threshold at 500% increase in homicides, with controls: -0.308 (-0.788 ; 0.172)

**Figure S3.** Average treatment effect on the treated (ATT) of increases in homicide rates by 100% and 500% on the total fertility rate, by period of first exposure.


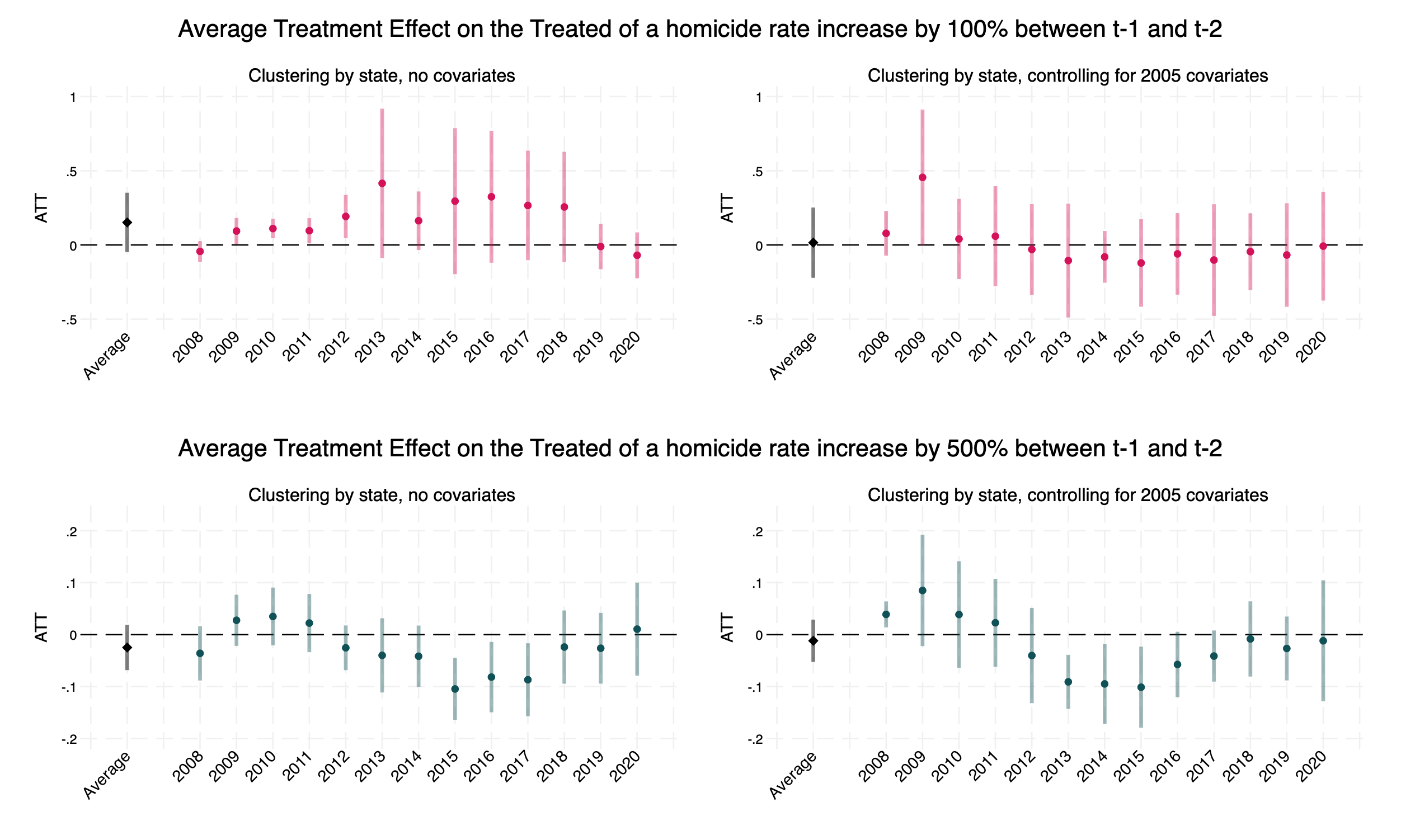


**Figure S4.** Average treatment effect on the treated (ATT) of increases in homicide rates by 100% and 500% on the crude birth rate, by period of first exposure.


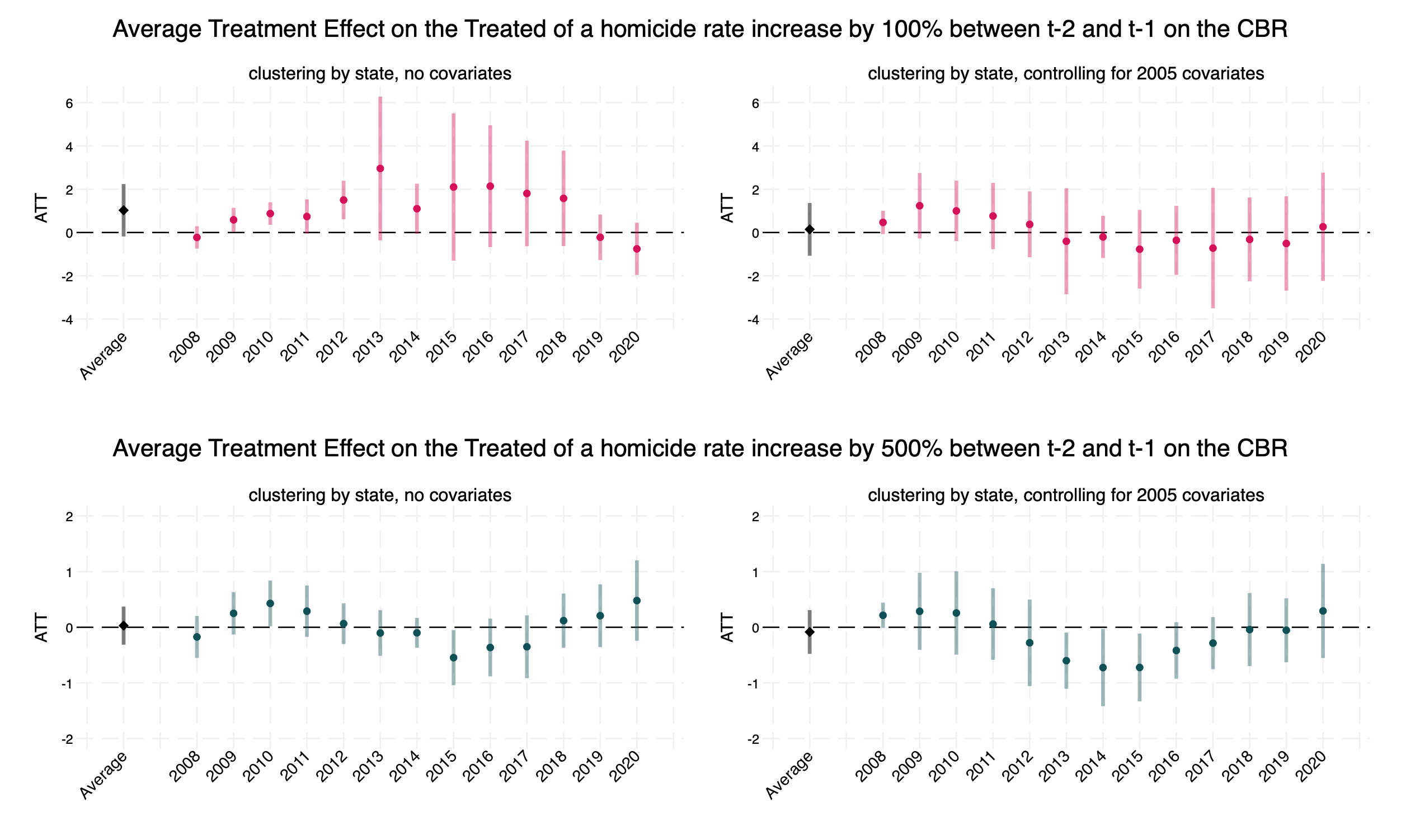


# ***E. Results from Random and Fixed-Effects Models for Fertility Desires without Municipality Time Trends***

**Table S6.** Coefficients and standard errors from random intercept and fixed-effects models for fertility desires.

|  | **Random intercept model** | **Fixed-effects model** |
| --- | --- | --- |
| Homicide rate, 3-month lag | -0.002 (0.000) *** | 0.000 (0.000) |
| Homicide rate, 6-month lag^a^ | -0.001 (0.000) *** | -0.001 (0.000) |
|  |  |  |
| Age (years) | 0.023 (0.002) *** | -0.032 (0.003) *** |
| Education: none |  |  |
| Secondary | -0.489 (0.033) *** | 0.034 (0.056) |
| High school | -0.573 (0.041) *** | 0.139 (0.071) |
| University | -0.698 (0.046) *** | 0.215 (0.084) * |
| Married: no |  |  |
| Yes | 0.319 (0.028) *** | 0.040 (0.038) |
| Working: no |  |  |
| < 30 hours/week | -0.043 (0.033) | 0.011 (0.036) |
| ≥ 30 hours/week | -0.145 (0.026) *** | 0.024 (0.031) |
| HH consumption | -0.001 (0.001) | 0.001 (0.001) |
| HH size | 0.067 (0.006) *** | -0.046 (0.011) *** |
| Health: poor |  |  |
| Fine | -0.066 (0.058) | -0.017 (0.063) |
| Good | -0.082 (0.059) | 0.002 (0.064) |
| Very good | -0.018 (0.069) | 0.123 (0.076) |
|  |  |  |
| State FE | yes | n/a |
|  |  |  |
| *N* (women) | 6,341 | 6,341 |
| *N* (observations) | 13,646 | 13,646 |

Notes: The random effects models include state fixed-effects; HH indicates “household.”

^a^ coefficient from an alternative model.

^†^ *p* < .10, * *p* < .05, ** *p* < .01, *** *p* < .001

**Table S7.** Coefficients and standard errors on the 3- and 6-month lag of the homicide rate from random-effects and fixed-effects models for fertility desires.

| **Panel A. By education** | | | | |
| --- | --- | --- | --- | --- |
|  | **Random intercept model** | | **Fixed effect model** | |
|  |  |  |  |  |
|  | **3-month lag** | **6-month lag** | **3-month lag** | **6-month lag** |
| **No formal education**  (*N* = 2,008, *n* = 4,452) | -0.001 (0.001) * | -0.001 (0.001) | -0.000 (0.001) | -0.000 (0.001) |
| **Secondary**  (*N* = 2,036, *n* = 4,481) | -0.002 (0.001) *** | -0.002 (0.000) *** | 0.000 (0.001) | -0.001 (0.001) |
| **High school**  (*N* = 1,196, *n* = 2,437) | -0.001 (0.001) | -0.001 (0.001) ^†^ | 0.001 (0.001) | -0.000 (0.001) |
| **University**  (*N* = 1,097, *n* = 2,273) | -0.002 (0.001) * | -0.001 (0.001) | -0.001 (0.001) | -0.001 (0.001) |
| **Panel B. By parity** | | | | |
|  | **Random intercept model** | | **Fixed effect model** | |
|  |  |  |  |  |
|  | **3-month lag** | **6-month lag** | **3-month lag** | **6-month lag** |
| **Childless**  (*N* = 2,184, *n* = 4,109) | -0.001 (0.001) | -0.000 (0.000) | 0.000 (0.001) | -0.000 (0.001) |
| **With children**  (*N* = 4,153, *n* = 9,534) | -0.002 (0.000) *** | -0.002 (0.000) *** | -0.000 (0.000) | -0.001 (0.000) * |
| **Panel C. By age group** | | | | |
|  | **Random intercept model** | | **Fixed effect model** | |
|  |  |  |  |  |
|  | **3-month lag** | **6-month lag** | **3-month lag** | **6-month lag** |
| **15–19**  (*N* = 1,164, *n* = 1,446) | 0.000 (0.001) | 0.000 (0.001) | 0.001 (0.004) | 0.003 (0.004) |
| **20–29**  (*N* = 3,289, *n* = 4,704) | -0.002 (0.001) *** | -0.001 (0.000) * | -0.000 (0.001) | -0.002 (0.001) * |
| **30–39**  (*N* = 3,219, *n* = 5,138) | -0.001 (0.001) | -0.002 (0.000) ** | 0.001 (0.001) | -0.001 (0.001) |
| **40–45**  (*N* = 1,756, *n* = 2,354) | -0.003 (0.001) * | -0.000 (0.001) | -0.002 (0.002) | 0.002 (0.002) |

Notes: Separate models fitted by educational attainment, parity, and age group. RE models include state fixed-effects.

^†^ *p* < .10, * *p* < .05, ** *p* < .01, *** *p* < .001

# ***F. Unabridged Tables Corresponding to Manuscript Table 4***

**Table S8.** Coefficients and standard errors from random effects models for fertility desires, by educational attainment.

| **Random effects model by educational attainment** | **No formal** | **Secondary** | **High School** | **University** |
| --- | --- | --- | --- | --- |
|  |  |  |  |  |
| Homicide rate 3-month lag | -0.001 | 0.000 | 0.000 | -0.001 |
|  | (0.001) | (0.001) | (0.001) | (0.001) |
| Homicide rate 6-month lag | 0.000 | -0.001 | 0.001 | -0.000 |
|  | (0.001) | (0.001) | (0.001) | (0.001) |
|  |  |  |  |  |
| Age | 0.093*** | 0.047*** | 0.034*** | 0.006 |
|  | (0.004) | (0.003) | (0.004) | (0.004) |
| Not married (ref) |  |  |  |  |
|  |  |  |  |  |
| Married | 0.492*** | 0.250*** | 0.080 | 0.322*** |
|  | (0.061) | (0.045) | (0.058) | (0.063) |
| Not working (ref) |  |  |  |  |
|  |  |  |  |  |
| Working < 30 hours/week | 0.038 | -0.076 | -0.160** | -0.102 |
|  | (0.058) | (0.042) | (0.054) | (0.056) |
| Working ≥ 30 hours/week | -0.017 | 0.000 | -0.061 | 0.042 |
|  | (0.066) | (0.055) | (0.077) | (0.067) |
| HH consumption | 0.002 | -0.004 | 0.023 | 0.001 |
|  | (0.004) | (0.003) | (0.016) | (0.002) |
| HH size | 0.141*** | 0.048*** | 0.054*** | 0.059*** |
|  | (0.012) | (0.010) | (0.014) | (0.015) |
| Health: poor (ref) |  |  |  |  |
|  |  |  |  |  |
| Fine | 0.033 | -0.060 | -0.065 | -0.001 |
|  | (0.088) | (0.099) | (0.164) | (0.227) |
| Good | 0.059 | -0.088 | -0.083 | -0.017 |
|  | (0.092) | (0.099) | (0.164) | (0.227) |
| Very good | 0.301* | 0.039 | -0.066 | 0.061 |
|  | (0.132) | (0.119) | (0.178) | (0.234) |
|  |  |  |  |  |
| Municipality $\times$ time | yes | yes | yes | yes |
|  |  |  |  |  |
| *N* (observations) | 4,451 | 4,481 | 2,437 | 2,273 |
| *N* (women) | 2,008 | 2,036 | 1,196 | 1,097 |

Notes: HH indicates “household;” the model includes municipal-level time trends; results correspond to those presented in Table 4, Panel A.

^†^ *p* < .10, * *p* < .05, ** *p* < .01, *** *p* < .001

**Table S9.** Coefficients and standard errors from fixed effects models for fertility desires, by educational attainment.

| **Fixed-effects model by educational attainment** | **No formal** | **Secondary** | **High School** | **University** |
| --- | --- | --- | --- | --- |
|  |  |  |  |  |
| Homicide rate, 3-month lag | -0.001 | 0.000 | -0.000 | 0.000 |
|  | (0.001) | (0.001) | (0.002) | (0.001) |
| Homicide rate, 6-month lag | -0.000 | -0.000 | -0.001 | 0.000 |
|  | (0.001) | (0.001) | (0.001) | (0.001) |
|  |  |  |  |  |
| Age | 0.011 | -0.025 | 0.004 | -0.010 |
|  | (0.022) | (0.021) | (0.036) | (0.027) |
| Not married (ref) |  |  |  |  |
|  |  |  |  |  |
| Married | 0.086 | 0.058 | -0.055 | 0.079 |
|  | (0.080) | (0.066) | (0.096) | (0.100) |
| Not working (ref) |  |  |  |  |
|  |  |  |  |  |
| Working < 30 hours/week | 0.106 | -0.006 | -0.066 | 0.079 |
|  | (0.066) | (0.053) | (0.073) | (0.075) |
| Working ≥ 30 hours/week | 0.007 | -0.037 | -0.033 | 0.098 |
|  | (0.070) | (0.064) | (0.094) | (0.085) |
| HH consumption | 0.001 | -0.002 | 0.030 | -0.000 |
|  | (0.004) | (0.003) | (0.022) | (0.002) |
| HH size | -0.066*** | -0.046* | -0.004 | -0.027 |
|  | (0.018) | (0.018) | (0.029) | (0.032) |
| Health: poor (ref) |  |  |  |  |
|  |  |  |  |  |
| Fine | 0.098 | -0.154 | -0.300 | -0.382 |
|  | (0.095) | (0.114) | (0.205) | (0.274) |
| Good | 0.094 | -0.186 | -0.267 | -0.298 |
|  | (0.100) | (0.116) | (0.207) | (0.276) |
| Very good | 0.404** | -0.090 | -0.204 | -0.270 |
|  | (0.141) | (0.139) | (0.226) | (0.284) |
|  |  |  |  |  |
| Municipality × time | yes | yes | yes | yes |
|  |  |  |  |  |
| *N* (observations) | 4,451 | 4,481 | 2,437 | 2,273 |
| *N* (women) | 2,008 | 2,036 | 1,196 | 1,097 |

Notes: HH indciates “household;” the model includes municipal-level time trends; results correspond to those presented in Table 4, Panel A.

^†^ *p* < .10, * *p* < .05, ** *p* < .01, *** *p* < .001

**Table S10.** Coefficients and standard errors from random effects models for fertility desires, by parity.

| **Random effects model by parity** | **Childless** | **With children** |
| --- | --- | --- |
|  |  |  |
| Homicide rate, 3-month lag | -0.000 | -0.000 |
|  | (0.001) | (0.001) |
| Homicide rate, 6-month lag | 0.000 | -0.000 |
|  | (0.001) | (0.000) |
| Age | -0.045*** | 0.080*** |
|  | (0.004) | (0.003) |
| Education: no formal (ref) |  |  |
|  |  |  |
| Secondary | -0.042 | -0.258*** |
|  | (0.059) | (0.037) |
| High school | 0.020 | -0.312*** |
|  | (0.063) | (0.052) |
| University | 0.121 | -0.527*** |
|  | (0.067) | (0.068) |
| Not married (ref) |  |  |
|  |  |  |
| Married | 0.040 | 0.212*** |
|  | (0.052) | (0.036) |
| Not working (ref) |  |  |
|  |  |  |
| Working < 30 hours/week | 0.045 | -0.099** |
|  | (0.040) | (0.032) |
| Working ≥ 30 hours/week | 0.031 | -0.042 |
|  | (0.059) | (0.038) |
| HH consumption | 0.017 | -0.000 |
|  | (0.012) | (0.001) |
| HH size | -0.011 | 0.134*** |
|  | (0.009) | (0.008) |
| Health: poor (ref) |  |  |
|  |  |  |
| Fine | -0.044 | 0.005 |
|  | (0.122) | (0.063) |
| Good | -0.043 | 0.013 |
|  | (0.122) | (0.065) |
| Very good | -0.006 | 0.156 |
|  | (0.132) | (0.080) |
|  |  |  |
| Municipality × time | yes | yes |
|  |  |  |
| *N* (observations) | 4,108 | 9,534 |
| *N* (women) | 2,184 | 4,153 |

Notes: HH indicates “household;” the model includes municipal-level time trends; results correspond to those presented in Table 4, Panel B.

^†^ *p* < .10, * *p* < .05, ** *p* < .01, *** *p* < .001

**Table S11.** Coefficients and standard errors from fixed effects models for fertility desires, by parity.

| **Fixed-effects model by parity** | **Childless** | **With children** |
| --- | --- | --- |
|  |  |  |
| Homicide rate, 3-month lag | 0.001 | -0.000 |
|  | (0.001) | (0.001) |
| Homicide rate, 6-month lag | -0.000 | -0.001 |
|  | (0.001) | (0.001) |
| Age | -0.011 | -0.007 |
|  | (0.022) | (0.014) |
| Education: no formal (ref) |  |  |
|  |  |  |
| Secondary | 0.103 | 0.030 |
|  | (0.145) | (0.062) |
| High school | 0.255 | 0.111 |
|  | (0.160) | (0.085) |
| University | 0.376* | 0.171 |
|  | (0.171) | (0.119) |
| Not married (ref) |  |  |
|  |  |  |
| Married | 0.044 | 0.069 |
|  | (0.092) | (0.045) |
| Not working (ref) |  |  |
|  |  |  |
| Working < 30 hours/week | 0.081 | 0.010 |
|  | (0.058) | (0.038) |
| Working ≥ 30 hours/week | 0.111 | -0.004 |
|  | (0.080) | (0.042) |
| HH consumption | 0.019 | 0.001 |
|  | (0.019) | (0.002) |
| HH size | -0.033 | -0.057*** |
|  | (0.022) | (0.013) |
| Health: poor (ref) |  |  |
|  |  |  |
| Fine | -0.258 | 0.025 |
|  | (0.162) | (0.069) |
| Good | -0.238 | 0.048 |
|  | (0.163) | (0.072) |
| Very good | -0.164 | 0.184* |
|  | (0.177) | (0.088) |
|  |  |  |
| Municipality × time | yes | yes |
|  |  |  |
| *N* (observations) | 4,108 | 9,534 |
| *N* (women) | 2,184 | 4,153 |

Notes: HH indicates “household;” the model includes municipal-level time trends; results correspond to those presented in Table 4, Panel B.

^†^ *p* < .10, * *p* < .05, ** *p* < .01, *** *p* < .001

**Table S12.** Coefficients and standard errors from random effects models for fertility desires, by age group.

| **Random-effects models by age group** | **15–19** | **20–29** | **30–39** | **40–45** |
| --- | --- | --- | --- | --- |
|  |  |  |  |  |
| Homicide rate, 3-month lag | 0.000 | -0.000 | 0.001 | -0.003* |
|  | (0.002) | (0.001) | (0.001) | (0.002) |
| Homicide rate, 6-month lag | 0.001 | 0.000 | -0.000 | -0.001 |
|  | (0.002) | (0.001) | (0.001) | (0.001) |
| Age | 0.043 | 0.014* | 0.081*** | 0.124*** |
|  | (0.024) | (0.006) | (0.006) | (0.017) |
| Education: no formal (ref) |  |  |  |  |
|  |  |  |  |  |
| Secondary | -0.063 | -0.126** | -0.326*** | -0.191* |
|  | (0.096) | (0.046) | (0.049) | (0.093) |
| High school | -0.045 | -0.137* | -0.436*** | -0.384** |
|  | (0.100) | (0.056) | (0.068) | (0.137) |
| University | -0.044 | -0.051 | -0.590*** | -0.686*** |
|  | (0.141) | (0.061) | (0.081) | (0.147) |
| Not married (ref) |  |  |  |  |
|  |  |  |  |  |
| Married | 0.064 | 0.289*** | 0.488*** | 0.509*** |
|  | (0.098) | (0.039) | (0.048) | (0.088) |
| Not working (ref) |  |  |  |  |
|  |  |  |  |  |
| Working < 30 hours/week | -0.033 | -0.037 | -0.071 | -0.162 |
|  | (0.084) | (0.039) | (0.044) | (0.084) |
| Working ≥ 30 hours/week | 0.110 | -0.043 | 0.027 | -0.028 |
|  | (0.122) | (0.055) | (0.053) | (0.099) |
| HH consumption | 0.003 | -0.000 | -0.000 | 0.004 |
|  | (0.029) | (0.006) | (0.002) | (0.014) |
| HH size | 0.018 | 0.010 | 0.197*** | 0.347*** |
|  | (0.015) | (0.007) | (0.011) | (0.019) |
| Health: poor (ref) |  |  |  |  |
|  |  |  |  |  |
| Fine | -0.989*** | 0.070 | -0.032 | 0.007 |
|  | (0.286) | (0.111) | (0.090) | (0.145) |
| Good | -1.022*** | 0.011 | -0.013 | 0.119 |
|  | (0.283) | (0.110) | (0.092) | (0.152) |
| Very good | -1.046*** | 0.040 | 0.067 | 0.367 |
|  | (0.298) | (0.123) | (0.115) | (0.199) |
|  |  |  |  |  |
| Municipality × time | yes | yes | yes | yes |
|  |  |  |  |  |
| *N* (observations) | 1,446 | 4,704 | 5,138 | 2,354 |
| *N* (women) | 1,164 | 3,289 | 3,219 | 1,756 |

Notes: HH indicates “household;” the model includes municipal-level time trends; results correspond to those presented in Table 4, Panel C.

^†^ *p* < .10, * *p* < .05, ** *p* < .01, *** *p* < .001

**Table S13.** Coefficients and standard errors from fixed effects models for fertility desires, by age group.

| **Fixed-effects model by age group** | **15–19** | **20–29** | **30–39** | **40–45** |
| --- | --- | --- | --- | --- |
|  |  |  |  |  |
| Homicide rate, 3-month lag | -0.009 | -0.001 | 0.001 | -0.005 |
|  | (0.012) | (0.001) | (0.001) | (0.003) |
| Homicide rate, 6-month lag | 0.007 | -0.002 | -0.001 | 0.001 |
|  | (0.011) | (0.001) | (0.001) | (0.003) |
| Age | -0.101 | 0.001 | 0.047 | -0.079 |
|  | (0.169) | (0.037) | (0.028) | (0.066) |
| Education: no formal (ref) |  |  |  |  |
|  |  |  |  |  |
| Secondary | 0.796* | 0.005 | 0.009 | 0.194 |
|  | (0.361) | (0.149) | (0.111) | (0.263) |
| High school | 0.890* | -0.041 | 0.086 | 0.076 |
|  | (0.356) | (0.190) | (0.148) | (0.393) |
| University | 0.885* | -0.075 | 0.008 | -0.101 |
|  | (0.424) | (0.246) | (0.192) | (0.487) |
| Not married (ref) |  |  |  |  |
|  |  |  |  |  |
| Married | -0.106 | 0.181 | 0.029 | -0.063 |
|  | (0.337) | (0.093) | (0.085) | (0.181) |
| Not working (ref) |  |  |  |  |
|  |  |  |  |  |
| Working < 30 hours/week | -0.143 | 0.102 | 0.049 | -0.014 |
|  | (0.200) | (0.072) | (0.063) | (0.156) |
| Working ≥ 30 hours/week | 0.137 | -0.032 | 0.034 | 0.038 |
|  | (0.272) | (0.091) | (0.068) | (0.170) |
| HH consumption | -0.043 | 0.004 | -0.001 | 0.018 |
|  | (0.059) | (0.007) | (0.002) | (0.018) |
| HH size | 0.118 | -0.048 | -0.045 | -0.075 |
|  | (0.078) | (0.025) | (0.027) | (0.048) |
| Health: poor (ref) |  |  |  |  |
|  |  |  |  |  |
| Fine | -0.128 | -0.189 | -0.180 | 0.247 |
|  | (0.611) | (0.187) | (0.123) | (0.219) |
| Good | -0.251 | -0.222 | -0.133 | 0.405 |
|  | (0.621) | (0.189) | (0.128) | (0.240) |
| Very good | -0.061 | -0.053 | -0.007 | 0.706* |
|  | (0.671) | (0.209) | (0.153) | (0.325) |
|  |  |  |  |  |
| Municipality × time | yes | yes | yes | yes |
|  |  |  |  |  |
| *N* (observations) | 1,446 | 4,704 | 5,138 | 2,354 |
| *N* (women) | 1,164 | 3,289 | 3,219 | 1,756 |

Notes: HH indicates “household;” the model includes municipal-level time trends; results correspond to those presented in Table 4, Panel C.

^†^ *p* < .10, * *p* < .05, ** *p* < .01, *** *p* < .001
